# Lifetime exposure to rubber dusts, fumes and N-nitrosamines and cancer mortality in a cohort of British rubber workers with 49 years follow-up

**DOI:** 10.1136/oemed-2018-105181

**Published:** 2019-02-16

**Authors:** Mira Hidajat, Damien Martin McElvenny, Peter Ritchie, Andrew Darnton, William Mueller, Martie van Tongeren, Raymond M Agius, John W Cherrie, Frank de Vocht

**Affiliations:** 1 Population Health Sciences, Bristol Medical School, University of Bristol, Bristol, UK; 2 Research Division, Institute of Occupational Medicine, Edinburgh, UK; 3 Statistics and Epidemiology Unit, Health and Safety Executive, Bootle, UK; 4 Centre for Occupational and Environmental Health, Centre for Epidemiology, School of Health Sciences, The University of Manchester, Manchester, UK; 5 Institute of Biological Chemistry, Biophysics and Bioengineering, Heriot Watt University, Edinburgh, UK

**Keywords:** rubber, occupational exposures, cohort, exposure-response, cancer

## Abstract

**Objectives:**

To quantitatively evaluate exposure-response associations between occupational exposures to rubber dust, fumes and N-nitrosamines and cancer mortality in the UK rubber industry.

**Methods:**

Competing risk survival analyses were used to examine cancer mortality risk in a cohort of 36 441 males aged 35+ years employed in the British rubber industry in 1967, followed up to 2015 (94% mortality). Exposure measurements are based on a population-specific quantitative job-exposure matrix for rubber dust, rubber fumes and N-nitrosamines from the EU-EXASRUB project.

**Results:**

Exposure (lifetime cumulative (LCE))-response associations were found for N-nitrosomorphiline and all cancers (subdistribution HR (SHR) 1.48, 95% CI 1.39 to 1.57) and cancers of the bladder, stomach, multiple myeloma, oesophagus, prostate and pancreas, as well as for N-nitrosodimethylamine and all cancers (SHR 2.08, 95% CI 1.96 to 2.21) and cancers of the bladder, stomach, leukaemia, multiple myeloma, prostate and liver. LCE to the N-nitrosamines sum were associated with increased risks from all cancers (SHR 1.89, 95% CI 1.78 to 2.01) and cancers of the lung, non-Hodgkin’s lymphoma and brain. LCE to rubber dust and fumes are associated with increased mortality from all cancers (rubber dust SHR 1.67, 95% CI 1.58 to 1.78; rubber fumes SHR 1.91, 95% CI 1.80 to 2.03) and cancers of the bladder, lung, stomach, leukaemia, multiple myeloma, non-Hodgkin’s lymphoma, oesophagus, prostate, pancreas and liver.

**Conclusions:**

Consistent with previous studies, N-nitrosamines exposures are associated with mortality from cancers of the bladder, lung, stomach, leukaemia, multiple myeloma, oesophagus, prostate, pancreas and liver. The long follow-up with nearly complete mortality enabled estimations of lifetime cancer mortality risk from occupational exposures in the rubber industry.

**Key messages**

**What is already known about this subject?**

- Employment in the rubber industry has been concluded to cause cancer by the International Agency for Research in Cancer.

**What are the new findings?**

- This paper updates exposure-response associations between cancer mortality and specific occupational exposures in the rubber industry for 49 years of follow-up and nearly complete mortality.
- Exposure-response associations were found for N-nitrosamines exposure and mortality from cancers of the bladder, stomach, oesophagus, leukaemia, multiple myeloma, prostate, pancreas and liver), and for rubber dust exposure and the lung.
- Elevated risks for cancer mortality without exposure-response patterns were also found for occupational exposures to rubber dust, rubber fumes and nitrosamines.
- Occupational exposure in the rubber industry was not found to be associated with laryngeal cancer.

**How might this impact on policy or clinical practice in the foreseeable future?**

- Results from this study contributed to the evidence of elevated cancer mortality risks from occupational exposures in the rubber industry by further clarifying the relationship between each carcinogen and cancer.

## Introduction

Employment in the rubber industry has been concluded to cause cancer by the International Agency for Research in Cancer (IARC). In particular, cancers of the bladder, lung, stomach, leukaemia and malignant lymphoma are designated as having sufficient evidence for excess incidence and/or mortality among workers in the rubber industry.^1^ Important carcinogenic exposures encountered in this industry are N-nitrosamines, rubber (process) dust, rubber fumes, polycyclic aromatic hydrocarbons including phthalates, aromatic amines including β-naphthylamine and solvents including benzene, among others.^2^

Exposures vary throughout the rubber manufacturing process. Rubber dust tends to have highest exposure in the beginning of the production process, particularly in handling raw materials. Rubber fumes (measured as the cyclohexane soluble fraction of rubber dust) and N-nitrosamines are generated during the heating and curing processes. Due to the complexity of exposure patterns and the numerous chemicals used in the rubber production process, disentangling exposure-response associations between specific suspected carcinogens and cancer risk in this industry remains difficult. Excess mortality from bladder cancer among rubber workers, particularly those employed before 1950, has been documented in multiple studies.^2–6^ Nevertheless, others found this association only for higher exposures to aromatic amines and not rubber dust.^7^ An exposure-response relationship between rubber dust and asbestos with lung cancer mortality was observed in Germany,^8^ but not in Poland.^7^ While some studies suggested possible associations between lung cancer mortality and N-nitrosamines exposure,^9^ others did not.^8 10 11^ Excess risk of stomach cancer has been found among workers early in the production line and in curing where rubber fumes exposure is highest, as well as those exposed to talc.^1^ Nevertheless, stomach cancer mortality was not associated with higher levels of inhalable aerosols^7^ or N-nitrosamines.^8 10 11^ Increased risk for leukaemia was associated with benzene and butadiene exposures,^1 12^ but mortality from leukaemia and other malignant lymphomas were not associated with inhalable aerosols among male rubber factory workers.^7^

IARC further reported limited, insufficient or inconsistent evidence for associations between rubber dust, rubber fumes and nitrosamines and several cancers.^1^ Oesophageal cancer mortality was found to have an exposure-response relationship with N-nitrosamines exposure.^8^ Non-statistically significant increases in brain and prostate cancer mortality were observed with higher exposures to N-nitrosamines.^8^ No evidence of associations between prostate and brain cancer mortality with rubber dust were found in the Polish industry.^7^ The relationship between pancreatic cancer and occupational exposure is unclear; small increased risks of incidence and mortality have been documented^13–15^ but not consistently.^7 16^ Laryngeal cancer mortality was found in excess in the highest exposure categories of rubber dust in Germany,^11^ but not in Poland.^7^ Evidence of an exposure-response association between liver cancer mortality and rubber dust was not statistically significant, possibly due to a low number of deaths.^7^ Possible explanations for these discrepancies may lie in differences in the industry and chemicals used across countries and time periods. Further difficulties include measurement error in the assessment of personal exposures over long periods of time.^1^

Previous studies in the British rubber industry have found similar trends of excess mortality or incidence of cancers of the bladder, particularly before 1949 due to exposures to 1-naphthylamine and 2-naphthylamine,^3^ lung due to exposures to rubber fumes^5^ and stomach as associated with rubber dust.^5^ Nevertheless, measurements of exposures to carcinogenic agents were only indirectly assessed through employment characteristics such as job title and work hours.

The current study aims to assess the specific association between cancer mortality and cumulative occupational exposures to rubber dust, rubber fumes and N-nitrosamines with follow-up of 49 years and a 94% mortality rate.

## Materials and methods

### Population

We used data from a cohort of male UK rubber factory workers aged 35 years or older as of 1 February 1967 (n=36 443 from 381 factories) followed up for mortality to December 2015. The median employment start year was 1954 (mean=1951.8, IQR 1946–1961). The cohort was set up by the predecessor to UK Health and Safety Executive.^4 5 17^

### Exposure assessment

Exposure assessment was based on estimates from the EU-EXASRUB database of measurements of compounds in rubber factories in Europe.^18^ Linear mixed effects models with random factory intercepts were used to estimate average exposure in each year between 1915 and 2000 and build a job-exposure matrix for rubber dusts, rubber fumes and N-nitrosamines.^19^ N-nitrosamines included in this study are N-nitrosodimethylamine (NDMA) and N-nitrosomorpholine (NMor) and N-nitrosamines sum score (NSS), a sum of NDMA, NMor, N-nitrosodibutylamine (NDBA), N-nitrosodiethylamine and N-nitrosopiperidine. Because only job information in 1967 was available, the primary analyses assumed all subjects remained in the same factory department (ie, not necessarily in the same job) throughout their careers and were employed until retirement at age 70, death or emigration. Lifetime cumulative exposures (LCE) to rubber dust, rubber fumes and N-nitrosamines were calculated for each worker based on the assumed number of years worked and department. Sensitivity analyses using different backcasting assumptions of constant average exposure level from the first year of available measurement were shown to have only minimal impact on individual cumulative exposure estimates (data not shown).

### Statistical methods

To examine the probability of dying from specific causes in a cohort with nearly complete mortality (94.1%), competing risk survival analysis was used to model time to death either from specific cancers, a competing event (death by another cause) or censored due to attrition (such as through emigration). Following the method by Fine and Gray,^20^ the current model is specified as:

$$\lambda_{k}^{*}\left(t\right)=\;\lim_{\Delta t\to 0}\frac{P\left(t\leq T<t+\Delta t,\;D=k|T\geq t\cup \left\{T<t,D\neq k\right\}\right)}{\Delta t}$$

where $\lambda_{k}^{*}\left(t\right)$ is the subdistribution hazard^20^ of cause *k* at time *t*, *T* is time of the first observed event and *D* is a random variable denoting type of event occurring. Subjects who experienced a competing event before the event of interest remain in the risk set and are weighted using the inverse probability of censoring weighting approach.^21 22^ This is in contrast to a standard Cox proportional hazard approach which would consider deaths from competing risks to be censored and would be removed from the risk set. Censoring competing events violates the assumption that censoring occurred at random and is independent from the risk of dying from the cause of death of interest, leading to a biased Kaplan-Meier estimator.^23^ Furthermore, within the context of competing risks, the interpretation of HRs from a standard Cox proportional hazard approach changes to the hazards of dying *if no other deaths occurred*,^21^ which is untenable in a cohort with 94.1% mortality rate. Subdistribution HRs (SHRs) are estimated using *stcrreg* in Stata V.15^24^ and comparable in interpretation to proportional HRs in Cox models.^25^

Cumulative exposure was divided into four quartiles and as a continuous metric to assess linearity of the exposure-response association. Outcomes are mortality from cancers previously associated with the rubber industry^1^: all cancers, cancers of the bladder, lung, stomach, multiple myeloma, leukaemia, larynx, oesophagus, prostate, non-Hodgkin’s lymphoma, pancreas, brain, liver and in situ, benign or unknown behaviour neoplasms.

Analyses were adjusted for birth year and LCE (in quartiles) to rubber dust, rubber fumes or N-nitrosamines. Because the baseline mean age of the sample is 50.1 years and median ages at death from cancer among males are between ages 60 and 75 years^26^, or 10 and 25 years after the baseline, and a lag or latency period between exposure and cancer development has been reported elsewhere,^27–31^ the analyses used 15-year lags, approximately around the midpoint of median cancer deaths.^26^ Analyses without lags for multiple myeloma, non-Hodgkin’s lymphoma and leukaemia deaths were tested and found lower effect sizes but similar directions and p values to lagged analyses.

Sensitivity analyses were conducted with alternative simulated employment durations based on information on employment durations from another, partly overlapping, cohort of British rubber factory workers.^4 5 32^ Ten simulated employment durations for each worker were generated from log-normal distributions (mean=2.355, SD=0.470) that for the whole sample had similar characteristics to this cohort; the 47th percentile of the distribution have 10 years of employment and 88th percentile have 18 or more years. Log-normal distributions were chosen over other functional forms because they provided the best fit to these parameters. Lifetime cumulative exposures were subsequently recalculated and competing risk survival analyses were performed for each of the 10 simulated employment durations to allow for assessment of the variability in the SHRs for the LCEs. Sensitivity analyses were conducted for all cancers and cancers of the bladder, lung, stomach, myeloma and leukaemia. Results of the primary analyses were mostly supported by the sensitivity analyses, including for all malignant neoplasms for all agents.

## Results

The cohort included 36 441 male workers in the UK rubber industry followed from 1967 to 2015 with 880 794 person years of time at risk and under observation. International Statistical Classification of Diseases and Related Health Problems codes and number of deaths are provided in OSM. We describe risk patterns as absence of exposure-response, linear (in log-space) exposure-response (increasing risk in quartiles (Q) 1–4), plateauing exposure-response (increasing risk in Q1–3, but plateau or reduction in Q4), and increased risk without an exposure-response pattern. Bladder cancer mortality represented 4.7% of all cancer deaths (n=417). The primary analyses (tables 1–2) showed a linear exposure-response relationship for NDMA (SHRs up to 2.82 (Q4) and NMor (SHRs up to 2.59 (Q4), plateauing exposure-response for NSS (SHRs up to 2.19 (Q3)) and increased risks without exposure-response pattern for rubber dust (SHRs up to 2.56 (Q4)). Sensitivity analyses (online suplementary materials) supported the primary analyses for all exposures. However, results for Q2 were not fully supported by the sensitivity analyses for NDMA where SHRs ranged from 1.14 (95% CI 0.85 to 1.52) to 1.56 (95% CI 1.18 to 2.06), and for NSS, where SHRs ranged from 1.26 (95% CI 0.95 to 1.68) to 1.51 (95% CI 1.15 to 1.99). Lung cancer mortality comprised the largest proportion of cancer deaths in the cohort at 37.1% (n=3377). A linear exposure-response relationship was found for exposures to rubber dust (SHRs up to 1.44 (Q4)). Sensitivity analyses supported this exposure-response pattern where Q4 SHRs ranged from 1.21 (95% CI 1.10 to 1.33) to 1.27 (95% CI 1.15 to 1.39), which is within the 95% CI of the primary analysis (1.31 to 1.58). Increased risks were found for NDMA exposure (SHRs up to 1.70 (Q4)). Plateauing exposure-response was observed for rubber fumes (SHRs up to 1.55 (Q3)), NSS (SHRs up to 1.60 (Q3)) and NMor (SHRs 1.19 (Q2 and Q3)). Sensitivity analyses support the exposure-response patterns observed for the primary analyses (OSM). Other differences between SHRs in the sensitivity and primary analyses were minimal with the largest difference found was for NDMA Q4 where primary analysis SHR=1.70 (95% CI: 1.41 to 2.10) and sensitivity analyses SHRs range from 1.40 (95% CI 1.27 to 1.54) to 1.46 (95% CI 1.32 to 1.60).

10.1136/oemed-2018-105181.supp1 Supplementary data

**Table 1:** Risk of death from selected cancers by 15-year lagged cumulative exposure to rubber dust and rubber fumes in a cohort of rubber factory workers in the UK

Malignancy	N	Cumulative rubber dust (year* mg/m^3^)			Cumulative rubber fumes (year* mg/m^3^)		
		Exposure*	SHR†	95% CI	Exposure‡	SHR†	95% CI
All malignant neoplasms	9101	I			I		
		II	1.39	1.31 to 1.47	II	1.38	1.3 to 1.46
		III	1.67	1.58 to 1.78	III	1.91	1.8 to 2.03
		IV	1.65	1.58 to 1.75	IV	1.66	1.57 to 1.76
P for trend			<0.01			<0.01	
Bladder	417	I			I		
		II	1.6	1.19 to 2.15	II	1.43	1.07 to 1.92
		III	2.49	1.89 to 3.29	III	3.12	2.38 to 4.09
		IV	2.56	1.95 to 3.37	IV	2.24	1.71 to 2.95
P for trend			0.6			0.98	
Lung	3377	I			I		
		II	1.3	1.19 to 1.43	II	1.16	1.06 to 1.27
		III	1.34	1.22 to 1.48	III	1.55	1.41 to 1.71
		IV	1.44	1.31 to 1.58	IV	1.52	1.39 to 1.66
P for trend			<0.01			<0.01	
Stomach	768	I			I		
		II	1.32	1.08 to 1.6	II	1.45	1.2 to 1.74
		III	1.78	1.47 to 2.15	III	1.75	1.43 to 2.15
		IV	1.59	1.3 to 1.93	IV	1.56	1.28 to 1.88
P for trend			0.94			0.83	
Leukaemia	195	I			I		
		II	1.29	0.85 to 1.96	II	1.32	0.88 to 1.96
		III	2.42	1.66 to 3.52	III	2.1	1.42 to 3.09
		IV	2.41	1.66 to 3.51	IV	2.1	1.45 to 3.05
P for trend			<0.01			<0.01	
Multiple myeloma	462	I			I		
		II	1.4	1.08 to 1.8	II	1.48	1.15 to 1.91
		III	1.99	1.55 to 2.55	III	2.12	1.65 to 2.73
		IV	1.8	1.39 to 2.34	IV	1.81	1.41 to 2.33
P for trend			0.27			0.04	
Non-Hodgkin’s lymphoma	141	I			I		
		II	1.51	0.97 to 2.35	II	1.66	1.04 to 2.64
		III	1.67	1.06 to 2.64	III	2.27	1.43 to 3.58
		IV	1.21	0.72 to 2.02	IV	1.58	0.97 to 2.57
P for trend			0.65			0.98	
Oesophagus	333	I			I		
		II	1.81	1.34 to 2.45	II	2.03	1.49 to 2.77
		III	2.26	1.67 to 3.08	III	2.55	1.85 to 3.51
		IV	2.01	1.46 to 2.76	IV	2.23	1.62 to 3.07
P for trend			0.25			0.02	
Prostate	885	I			I		
		II	2.25	1.8 to 2.81	II	2.62	2.1 to 3.28
		III	3.37	2.73 to 4.16	III	4.03	3.26 to 4.99
		IV	2.96	2.38 to 3.67	IV	2.84	2.27 to 3.55
P for trend			0.26			0.04	
Larynx	62	I			I		
		II	0.95	0.45 to 2.02	II	1.11	0.58 to 2.11
		III	1.67	0.87 to 3.2	III	1.11	0.52 to 2.33
		IV	1.73	0.91 to 3.27	IV	1.47	0.78 to 2.76
P for trend			0.69			0.5	
Brain	106	I			I		
		II	1.06	0.64 to 1.77	II	1.31	0.78 to 2.2
		III	2.22	1.39 to 3.53	III	1.22	0.67 to 2.2
		IV	0.51	0.23 to 1.13	IV	1.53	0.91 to 2.59
P for trend			<0.01			0.06	
Pancreas	328	I			I		
		II	1.45	1.07 to 1.96	II	1.95	1.45 to 2.62
		III	2	1.49 to 2.69	III	2.48	1.82 to 3.38
		IV	1.93	1.42 to 2.62	IV	1.83	1.33 to 2.51
P for trend			0.07			0.02	
Liver	122	I			I		
		II	2.86	1.67 to 4.89	II	2.02	1.21 to 3.39
		III	3.12	1.81 to 5.35	III	3.35	2.05 to 5.46
		IV	3.4	1.96 to 5.89	IV	2.31	1.36 to 3.94
P for trend			0.24			0.3	
In situ, benign and tumours of unknown origin	36	I			I		
		II	0.17	0.02 to 1.24	II	0.67	0.23 to 1.98
		III	0.72	0.22 to 2.37	III	1.28	0.37 to 4.38
		IV	1.67	0.71 to 3.92	IV	1.21	0.49 to 3.02
P for trend			0.01			0.06	
Cancers associated with the rubber industry§	6860	I			I		
		II	1.38	1.29 to 1.47	II	1.36	1.27 to 1.45
		III	1.69	1.58 to 1.81	III	1.88	1.75 to 2.01
		IV	1.63	1.53 to 1.74	IV	1.65	1.55 to 1.77
P for trend			<0.01			<0.01	

*Exposure quartiles I: <9.50 year mg/m^3^ (400 269.3 person years); II: 9.50–16.68 year mg/m^3^ (197 237.6 person years); III: 16.68–27.03 year mg/m^3^ (145 564.8 person years); IV: >27.03 year mg/m^3^ (137 631.9 person years).

†SHRs from competing risk survival analysis adjusted for birth year.

‡Exposure quartiles I: <2.98 year mg/m^3^ (395 023.3 person years); II: 2.98–5.55 year mg/m^3^ (204 533.3 person years); III: 5.55–9.36 year mg/m^3^ (130 505 person years); IV: >9.36 year mg/m^3^ (150 732 person years).

§Cancers of the bladder, lung, stomach, oesophagus, prostate, larynx, brain, pancreas, liver, lymphatic and haematopoietic tissue.

SHR, subdistribution HR.

**Table 2:** Risk of death from selected cancers by 15-year lagged cumulative exposure to nitrosamines sum score, NDMA and NMor in a cohort of rubber factory workers in the UK

Malignancy	N	Cumulative nitrosamines sum score* (year* μg/m^3^)			Cumulative NDMA (year* μg/m^3^)			Cumulative NMor (year* μg/m^3^)		
		Exposures†	SHR‡	95% CI	Exposures§	SHR‡	95% CI	Exposures¶	SHR‡	95% CI
All malignant neoplasms	9101	I			I			I		
		II	1.47	1.39 to 1.56	II	1.32	1.25 to 1.40	II	1.22	1.15 to 1.29
		III	1.89	1.78 to 2.01	III	1.83	1.72 to 1.95	III	1.44	1.36 to 1.53
		IV	1.49	1.41 to 1.58	IV	2.08	1.96 to 2.21	IV	1.48	1.39 to 1.57
P for trend			<0.01			<0.01			<0.01	
Bladder	417	I			I			I		
		II	1.41	1.06 to 1.89	II	1.57	1.19 to 2.07	II	1.31	0.97 to 1.76
		III	2.19	1.69 to 2.84	III	2.45	1.87 to 3.21	III	1.67	1.25 to 2.24
		IV	1.95	1.51 to 2.52	IV	2.82	2.16 to 3.67	IV	2.59	1.99 to 3.38
P for trend			0.01			<0.01			<0.01	
Lung	3377	I			I			I		
		II	1.21	1.10 to 1.34	II	1.21	1.10 to 1.32	II	1.06	0.97 to 1.16
		III	1.6	1.45 to 1.76	III	1.54	1.39 to 1.70	III	1.19	1.09 to 1.31
		IV	1.36	1.25 to 1.49	IV	1.7	1.54 to 1.87	IV	1.19	1.08 to 1.31
P for trend			0.03			0.36			<0.01	
Stomach	768	I			I			I		
		II	1.61	1.32 to 1.95	II	1.32	1.10 to 1.57	II	1.28	1.06 to 1.55
		III	1.78	1.46 to 2.17	III	1.62	1.32 to 1.98	III	1.43	1.17 to 1.75
		IV	1.37	1.13 to 1.65	IV	1.72	1.41 to 2.10	IV	1.49	1.22 to 1.81
P for trend			0.1			0.01			0.01	
Leukaemia	195	I			I			I		
		II	2.6	1.74 to 3.91	II	1.52	0.99 to 2.33	II	1.45	0.97 to 2.16
		III	3.08	2.02 to 4.71	III	3.27	2.20 to 4.86	III	1.46	0.95 to 2.23
		IV	2.08	1.38 to 3.14	IV	3.47	2.35 to 5.13	IV	1.96	1.32 to 2.91
P for trend			0.02			<0.01			<0.01	
Multiple myeloma	462	I			I			I		
		II	2.09	1.61 to 2.71	II	1.59	1.22 to 2.08	II	1.48	1.15 to 1.91
		III	2.35	1.80 to 3.06	III	2.78	2.15 to 3.60	III	1.47	1.12 to 1.92
		IV	1.79	1.38 to 2.32	IV	2.81	2.17 to 3.64	IV	1.82	1.40 to 2.36
P for trend			0.02			<0.01			<0.01	
Non-Hodgkin’s lymphoma	141	I			I			I		
		II	1.82	1.11 to 2.96	II	1.51	0.93 to 2.43	II	1.24	0.79 to 1.96
		III	2.24	1.42 to 3.53	III	2.17	1.35 to 3.47	III	1.58	1.01 to 2.46
		IV	1.54	0.95 to 2.48	IV	2.25	1.41 to 3.59	IV	1.36	0.83 to 2.24
P for trend			0.55			0.11			0.23	
Oesophagus	333	I			I			I		
		II	2.14	1.56 to 2.93	II	1.7	1.24 to 2.33	II	1.74	1.28 to 2.37
		III	2.42	1.75 to 3.33	III	2.43	1.78 to 3.31	III	1.86	1.35 to 2.56
		IV	2.01	1.46 to 2.75	IV	3.04	2.26 to 4.09	IV	2.25	1.64 to 3.08
P for trend			0.26			0.26			<0.01	
Prostate	885	I			I			I		
		II	3.05	2.46 to 3.78	II	2.32	1.82 to 2.97	II	1.97	1.59 to 2.45
		III	3.75	3.02 to 4.64	III	4.87	3.89 to 6.11	III	2.57	2.08 to 3.18
		IV	2.49	2.00 to 3.11	IV	5.36	4.27 to 6.73	IV	2.71	2.20 to 3.34
P for trend			0.02			<0.01			<0.01	
Larynx	62	I			I			I		
		II	1.32	0.67 to 2.58	II	1.71	0.93 to 3.14	II	1.78	0.93 to 3.39
		III	1.99	1.08 to 3.65	III	1.49	0.69 to 3.19	III	1.49	0.73 to 3.00
		IV	0.63	0.28 to 1.40	IV	1.39	0.67 to 2.90	IV	1.3	0.62 to 2.71
P for trend			0.37			0.24			0.4	
Brain	106	I			I			I		
		II	1.16	0.65 to 2.08	II	1.3	0.76 to 2.25	II	2.16	1.21 to 3.87
		III	1.1	0.60 to 2.00	III	1.26	0.68 to 2.36	III	3.16	1.79 to 5.55
		IV	1.75	1.06 to 2.90	IV	2.5	1.53	IV	2.34	1.22 to 4.47
P for trend			0.03			<0.01			<0.01	
Pancreas	328	I			I			I		
		II	2.2	1.63 to 2.97	II	1.59	1.18 to 2.15	II	1.32	0.98 to 1.78
		III	2.15	1.56 to 2.96	III	2.19	1.60 to 3.00	III	1.44	1.05 to 1.99
		IV	1.77	1.31 to 2.39	IV	2.6	1.94 to 3.49	IV	1.96	1.46 to 2.64
P for trend			0.93			0.42			<0.01	
Liver	122	I			I			I		
		II	2.16	1.28 to 3.66	II	1.53	0.93 to 2.50	II	1.17	0.68 to 2.00
		III	3.2	1.97 to 5.19	III	1.96	1.16 to 3.29	III	1.91	1.16 to 3.14
		IV	1.6	0.94 to 2.73	IV	2.86	1.78 to 4.59	IV	1.84	1.11 to 3.05
P for trend			0.52			0.03			<0.01	
In situ, benign and tumours of unknown origin	36	I			I			I		
		II	1.18	0.38 to 3.63	II	0.78	0.31 to 1.97	II	0.42	0.14 to 1.21
		III	1.05	0.36 to 3.07	III	0.46	0.06 to 3.67	III	0	0.00 to 0.00
		IV	0.27	0.06 to 1.13	IV	0.3	0.04 to 2.18	IV	0.53	0.18 to 1.53
P for trend			0.36			0.4			0.07	
Cancers associated with the rubber industry**	6860	I			I			I		
		II	1.47	1.37 to 1.57	II	1.32	1.23 to 1.41	II	1.21	1.13 to 1.29
		III	1.85	1.73 to 1.98	III	1.82	1.70 to 1.95	III	1.39	1.29 to 1.48
		IV	1.49	1.40 to 1.59	IV	2.07	1.94 to 2.21	IV	1.49	1.39 to 1.59
P for trend			<0.01			<0.01			<0.01	

*Cumulative nitrosamines sum score is a sum of NDMA, NMor, NDBA, NDEA and NPIP.

†Exposure quartiles I: <10.03 year µg/m^3^ (384 893.5 person years); II: 10.03–21.38 year µg/m^3^ (165 784.3 person years); III: 21.38–442.93 year µg/m^3^ (142 507.6 person years); IV: >442.93 year µg/m^3^ (187 608.3 person years).

‡SHRs from competing risk survival analysis adjusted for birth year.

§Exposure quartiles I: <3.12 year µg/m^3^ (407 378.9 person years); II: 3.12–5.96 year µg/m^3^ (210 818.3 person years); III: 5.96–9.67 year µg/m^3^ (133 681.9 person years); IV: >9.67 year µg/m^3^ (128 914.5 person years).

¶Exposure quartiles I: <4.69 year µg/m^3^ (360 154.8 person years); II: 4.69–9.77 year µg/m^3^ (216 870.8 person years); III: 9.77–16.40 year µg/m^3^ (165 740.5 person years); IV: >16.40 year µg/m^3^ (138 027.5 person years).

**Cancers of the bladder, lung, stomach, oesophagus, prostate, larynx, brain, pancreas, liver, lymphatic and haematopoietic tissue.

NDBA, N-nitrosodibutylamine; NDEA, N-nitrosodiethylamine; NDMA, N-nitrosodimethylamine; NMor, N-nitrosomorpholine; NPIP, N-nitrosopiperidine; SHR, subdistribution HR.

For stomach cancer mortality (n=768, 8.4% of all cancer deaths), a linear exposure-response relationship was found for exposures to NDMA (SHRs up to 1.72 (Q4)) and NMor (SHRs up to 1.49 (Q4)). However, this exposure-response relationship was not supported by the sensitivity analyses. Rather, only elevated SHRs for Q3 (NDMA, NMor) and Q4 (NMor) were consistently found across the simulations. Increased risks were observed for rubber dust (SHR up to 1.78 Q3)), rubber fumes (SHRs up to 1.75 (Q3)) and NSS (SHRs up to 1.78 (Q3)). This was supported by the sensitivity analyses.

A linear exposure-response association was found for leukaemia mortality (n=195, 2.1% of all cancer deaths) and NDMA exposures (p for trend <0.001) (SHRs up to 3.47 (Q4)). Plateauing exposure-response were found for rubber dust (SHRs up to 2.42 (Q3)) and NSS (SHRs up to 3.08 (Q3)). Increased risks were found for rubber fumes (SHRs up to 2.10 (Q3 and Q4)) and NMor (SHRs up to 1.96 (Q4)). Sensitivity analyses results show consistently elevated and statistically significant SHRs for Q3 for NSS and Q4 for rubber dust, rubber fumes and NMor.

A linear exposure-response relationship was found for multiple myeloma mortality (n=462, 5.1% of all cancer mortality) with exposures to NDMA (SHRs up to 2.81 (Q4), p for linear trend <0.01) and NMor (SHRs up to 1.82 (Q4), p for linear trend <0.01). Sensitivity analyses support this pattern. Plateauing exposure-response was found for rubber fumes (SHRs up to 2.12 (Q3), p=0.04 for trend) and NSS (SHRs up to 2.35 (Q3), p=0.02 for trend). Increased risks were found for rubber dust (SHRs up to 1.99 (Q4)). Sensitivity analyses support the patterns for NSS and rubber fumes exposures and partially support NMor (Q4) and rubber dust (Q3 and Q4).

Increased risks for non-Hodgkin’s lymphoma mortality (n=141, 1.5% of all cancer mortality) were observed for rubber dust (SHR up to 1.68 (Q3)), rubber fumes (SHRs up to 2.27 (Q3)), NSS (SHRs up to 2.24 (Q3)), NDMA (SHRs up to 2.26 (Q4)) and NMor (SHRs up to 1.58 (Q3)).

A linear exposure-response relationship for oesophageal cancer mortality (n=333, 3.7% of all cancer deaths) as found for NMor (SHRs up to 2.25 (Q4), p<0.001 for trend) and plateauing exposure-response relationship was found for rubber fumes (SHRs up to 2.55 (Q3)). Increased risks were found for rubber dust (SHRs up to 2.26 (Q3)), NSS (SHRs up to 2.42 (Q3)) and NDMA (SHRs up to 3.04 (Q4)).

Linear exposure-response relationships between prostate cancer mortality (n=885, 9.7% of all cancer deaths) and exposures to NDMA (SHRs up to 5.36 (Q4)) and NMor (SHRs up to 2.71 (Q4)) were found. Plateauing exposure-response were found for rubber fumes (SHRs up to 4.03 (Q3)) and NSS (SHRs up to 3.75 (Q3)). Increased risks were found for rubber dust (SHRs up to 3.37 (Q3)).

Results for laryngeal cancer mortality (n=62, 0.7% of all cancer deaths) show elevated SHRs for all exposures, but did not achieve statistical significance, except for NSS (Q3).

Results for brain cancer mortality (n=106, 1.2% of all cancer deaths) show plateauing exposure-response relationships for NMor (SHRs up to 3.16 (Q3)), elevated SHRs in Q3 for rubber dust (SHR 2.22) and Q4s for NSS (SHR 1.75) and NDMA (SHR 2.50).

A linear exposure-response relationship was found for pancreatic cancer mortality (n=328, 3.6% of all cancer deaths) and NMor (SHRs up to 1.96 (Q4)). Plateauing exposure-response relationship was found for rubber fumes (SHRs up to 2.48 (Q3)) and increased risks without a trend were found for rubber dust (SHRs up to 2.00 (Q3)), NDMA (SHRs up to 2.60 (Q4)) and NSS (SHRs up to 2.20 (Q2)).

A linear exposure-response relationship between liver cancer mortality (n=122, 1.3% of all cancer deaths) and exposures to NDMA (SHRs up to 2.86 (Q4)) were observed. Plateauing exposure-response was found for NMor exposure (SHRs up to 1.91 (Q3)). Increased risks were found for rubber dust (SHRs up to 3.40 (Q4)), rubber fumes (SHRs up to 3.35 (Q3)) and NSS (SHRs up to 3.20 (Q3)).

## Discussion

This study examined cancer mortality risks associated with occupational exposures to rubber dust, rubber fumes and nitrosamines in a large occupational cohort of UK rubber manufacturing workers with 49 years of follow-up (880 794 person years). We found increased risks associated with cumulative exposures to rubber dust, rubber fumes and N-nitrosamines, which can be attributed to the unique combination of very long follow-up of the cohort, 94% mortality and selection of the exposures based on prior knowledge on carcinogenicity.^1^

Linear exposure-response relationships were found for cumulative exposure to NDMA (bladder, stomach, leukaemia, multiple myeloma, prostate, liver), NMor (bladder, stomach, multiple myeloma, oesophagus, prostate, pancreas) and rubber dust (lung). Plateauing exposure-response relationships were found for rubber dust (leukaemia), rubber fumes (lung, leukaemia, multiple myeloma, oesophagus, prostate, pancreas), NSS (bladder, lung, leukaemia, multiple myeloma, prostate) and NMor (lung, brain, liver). Increased risks were found for rubber dust (bladder, stomach, multiple myeloma, non-Hodgkin’s lymphoma, oesophagus, prostate, pancreas, brain, liver), rubber fumes (bladder, stomach, leukaemia, non-Hodgkin’s lymphoma, liver), NSS (stomach, non-Hodgkin’s lymphoma, brain, liver), NDMA (lung, non-Hodgkin’s’ lymphoma, oesophagus, brain, pancreas) and NMor (leukaemia, non-Hodgkin’s lymphoma). Occupational exposures were not associated with laryngeal cancer mortality.

Exposure-response associations between occupational exposures to nitrosamines and mortality from oesophageal and prostate cancers have been reported previously.^8 11^ We additionally observed increased stomach cancer risks with NDMA and NMor, in contrast to several studies^8 11 ^and consistent with others.^10 33^

Although this study found exposure-response associations between lung cancer mortality and occupational exposure to rubber dust and elevated risks associated with rubber fumes, NSS, NDMA and NMor, several previous studies did not observe associations between lung cancer mortality and N-nitrosamine exposure^8 10 11^ and others found inconsistent evidence on associations with rubber dust.^7 8^ It is possible that these results are capturing effects of smoking on lung cancer mortality rather than occupational exposures because individual smoking histories were unavailable. To obtain some indication of the possible confounding effect of smoking in this cohort, we used a statistical external adjustment method.^34^ External Monte Carlo analyses based on information on smoking prevalence, ex-smokers and never smokers from a cohort of rubber industry entrants after 1982^18^ indicated that mean bias is only 1.6% compared with the general population. To achieve as much as 10% (attenuating) bias internally, 12%–14% more smokers and 12%–14% fewer never smokers would have to be present in Q4 compared with Q1 (data not shown), which seems unlikely; suggesting that confounding by smoking in this cohort was likely not a significant confounding factor.

There are several strengths to this study. First, the 49-year follow-up period constitutes the longest in cohort studies of rubber workers in the UK. With a nearly entirely deceased cohort, these analyses provide the most precise and complete lifetime risks from exposures encountered in the rubber manufacturing industry. Second, instead of qualitative information on jobs, exposure assessments from a quantitative job-exposure matrix^19^ based on historic exposure measurements previously collated in the EXASRUB database^18^ were used.

Aside from these strengths, several limitations exist in this study. Information regarding individual employment histories were unavailable before 1967 and during the follow-up period. As such, the main analysis assumed continuous employment until retirement at age 70, emigration or death. To address this limitation, we conducted sensitivity analyses with varying employment durations, which supported the main results of this study in all but a few instances. Differences observed between the results of the main analyses and the sensitivity analyses imply that results for low incidence cancers such as leukaemia (n=195) are more sensitive than high-incidence cancers such as lung cancer (n=3377) to the length of occupational exposure to each agent, which is further dependent on employment duration. Second, we assumed a 15-year lag between exposure and clinical manifestation of the cancer. However, these lags are not uniform across all cancers and are generally somewhat shorter for bloodborne cancers.^35^ Nonetheless, as an overall approximation 15 years seems appropriate. Third, although some cancers are less fatal than others and this study used underlying cause of death from the death certificate without any cancer incidence data, some cancers may have been undercounted and comparison to cancer mortality in the general population would yield lower rates. However, results for selected cancers from SMR analyses of the same data^33^ showed either higher cancer mortality or no difference from the general population. Fourth, information on important confounders such as smoking and other lifestyle factors were unavailable, although additional simulations indicated smoking was unlikely to be a significant confounding factor. Fifth, the cohort was subject to selection effects at recruitment because workers had to survive until age 35 to be included in the cohort. Sixth, the JEM provides estimates of average exposure for workers which introduces measurement error in individual assessments. However, group-based exposure assessment generally leads to Berkson-type rather than classical measurement error, which results in attenuation of exposure-response associations rather than biased results.^36^ Finally, cross-contamination between departments could not rule out the need for multipollutant models, but given the high correlations between exposures this requires different and complex statistical modelling, with currently unknown validity in this context.

## Conclusions

In summary, we examined the exposure-response association between occupational exposures to rubber dust, rubber fumes and nitrosamines with cancer mortality in a cohort of 36 441 UK rubber factory workers with a follow-up from 1967 to 2015. Consistent with previous studies, N-nitrosamines exposures in the rubber industry, were associated with mortality from cancers of the bladder, lung, stomach, leukaemia, multiple myeloma, oesophagus, prostate, pancreas and liver. We also found linear exposure-response associations where the highest exposures to NMor more than double the risks for mortality from cancers of the bladder, oesophagus and prostate, similarly to the highest exposures of NDMA and cancers of the bladder, leukaemia, multiple myeloma, prostate, liver. Linear exposure-response relationships resulting in modest increased risks were also found for exposures to NMor and cancers of the stomach, multiple myeloma and pancreas and NDMA with cancers of the stomach. Furthermore, exposures to rubber dust and rubber fumes were found to be associated with higher risks for these cancers as well. Results from this study contribute to the evidence of elevated cancer mortality risks from occupational exposures in the rubber industry by further clarifying the relationship between each carcinogen and cancer with implications for the industry today where occupational exposures to N-nitrosamines continues to persist, although at greatly reduced levels compared with several decades ago.
