# Correction: *Lifetime exposure to rubber dusts, fumes and N-nitrosamines and cancer mortality in a cohort of British rubber workers with 49 years follow-up*

**DOI:** 10.1136/oemed-2018-105181corr1

**Published:** 2022-08-11

Hidajat M, McElvenny DM, Ritchie P, *et al*. Lifetime exposure to rubber dusts, fumes and N-nitrosamines and cancer mortality in a cohort of British rubber workers with 49 years follow-up. *Occup Environ Med* 2020;77:316–23.

In the Discussion section on p. 257 third paragraph, starting ‘External Monte Carlo analyses based on information on smoking prevalence…’ reference #18 is incorrect; instead it should be reference #17, which points to the paper below:

Dost A, Straughan J, Sorahan T. A cohort mortality and cancer incidence survey of recent entrants 1982–91, to the UK rubber industry: findings for 1983–2004. *Occup Med* 2007;57:186–90.

