# Supplementary material for: Lifetime exposure to rubber dusts, fumes and N-nitrosamines and cancer mortality in a cohort of British rubber workers with 49 years follow-up
Source: Occup Environ Med. 2019 Feb 16;76(4):250–8. doi: 10.1136/oemed-2018-105181 (PMC6581114; doi:10.1136/oemed-2018-105181)
Supplement: Supplementary data [file oemed-2018-105181supp001.pdf]

Lifetime exposure to rubber dusts, fumes and N-Nitrosamines and cancer mortality in a cohort of British rubber workers with 49 years follow-up

Mira Hidajat[1], Damien McElvenny[2], Peter Ritchie[2], Andrew Darnton[3], William Mueller[2], Martie van Tongeren[4], Raymond Agius[4], John W Cherrie[2,5], Frank de Vocht[1]

[1] Population Health Sciences, Bristol Medical School, University of Bristol, Bristol, UK

[2] Research Division, Institute of Occupational Medicine, Edinburgh, UK

[3] Statistics and Epidemiology Unit, Health and Safety Executive, Bootle, UK

[4] Centre for Occupational and Environmental Health, Centre for Epidemiology, School of Health Sciences, The University of Manchester, Manchester, UK

[5] Institute of Biological Chemistry, Biophysics and –Bioengineering, Heriot Watt University, Edinburgh, UK

Online Supplement. Results of simulations by cancer and agent

A70 represent results for the main analysis where employment duration is continuous until retirement at age 70, emigration, or death

D1-10 represent results of simulations of employment durations based on a similar UK cohort[4,5,17,32]

X-axis represents Sub Hazard Ratios (SHR) for each quartile of exposure

Exposure quartiles

Rubber dust (I: <9.50 year mg/m^3; II: 9.50-16.68 year mg/m^3; III: 16.68-27.03 year mg/m^3; IV:>27.03 year mg/m^3)

Rubber fumes (I: <2.98 year mg/m^3 ; II: 2.98-5.55 year mg/m^3; III: 5.55-9.36 year mg/m^3; IV: >9.36 year mg/m^3

NSS (I: <10.03 year µg/m^3; II: 10.03-21.38 year µg/m^3; III: 21.38-442.93 year µg/m^3; IV:>442.93 year µg/m^3)

NDMA (I: <3.12 year µg/m^3; II: 3.12-5.96 year µg/m^3; III: 5.96-9.67 year µg/m^3; IV: >9.67 year µg/m^3)

NMor (I: <4.69 year µg/m^3; II: 4.69-9.77 year µg/m^3; III: 9.77-16.40 year µg/m^3; IV: >16.40 year µg/m^3)

BLADDER CANCER

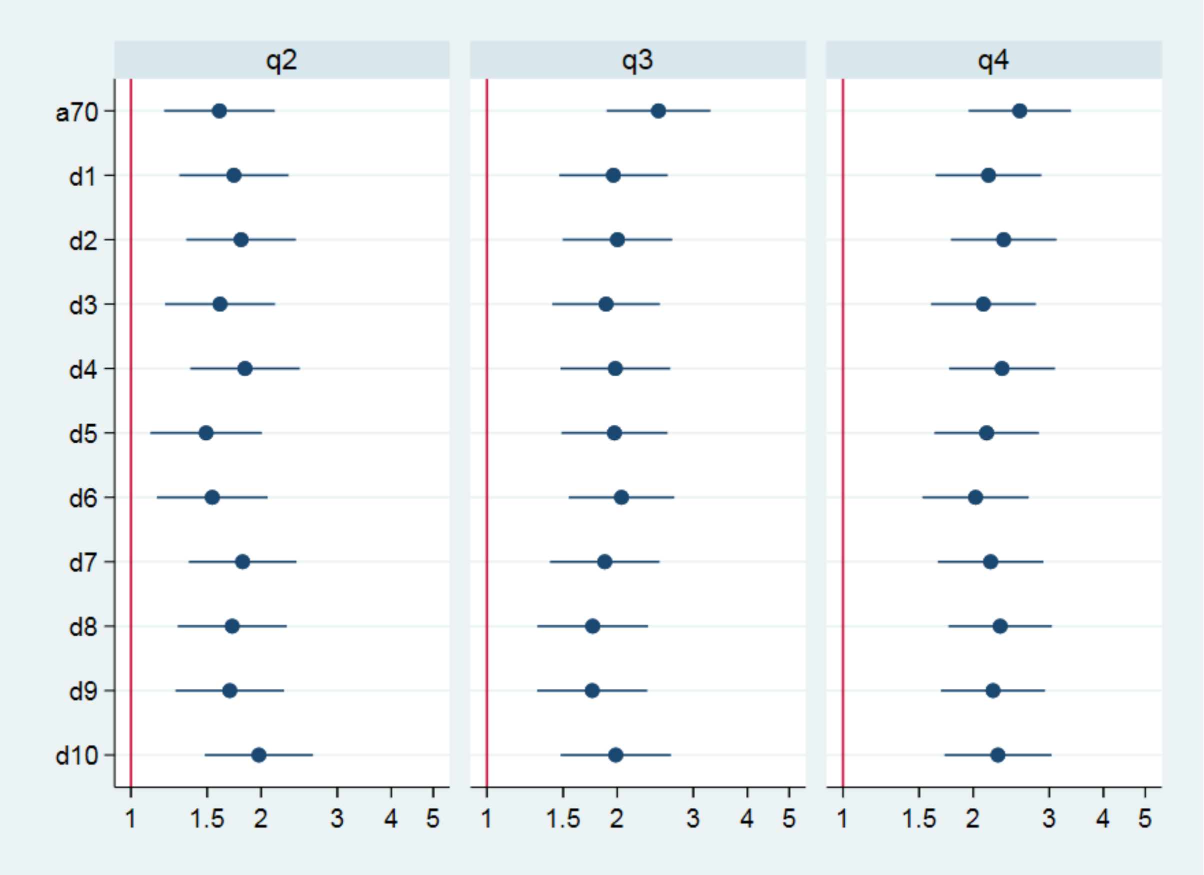

Figure 1. Sensitivity analyses of employment durations and exposure-response associations between bladder cancer mortality and lifetime cumulative exposure to rubber dust

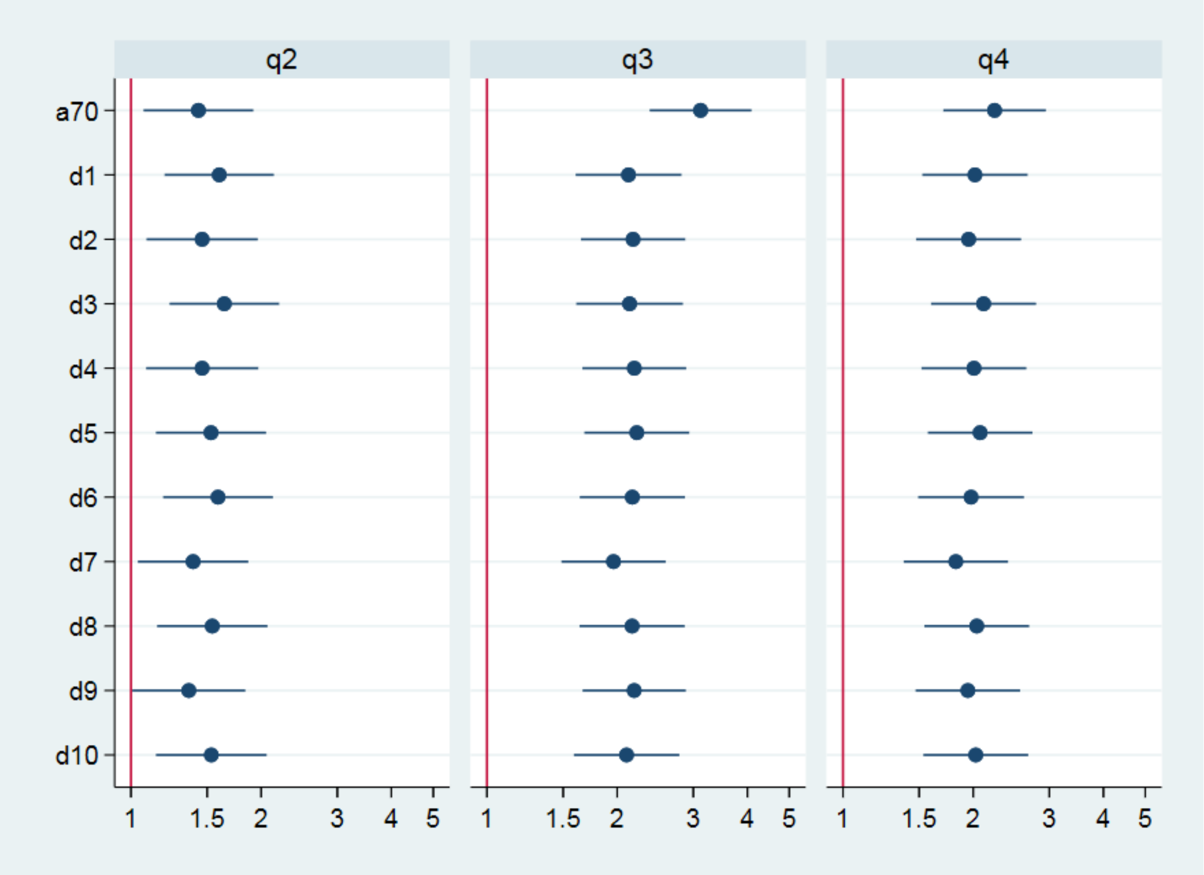

Figure 2. Sensitivity analyses of employment durations and exposure-response associations between bladder cancer mortality and lifetime cumulative exposure to rubber fumes

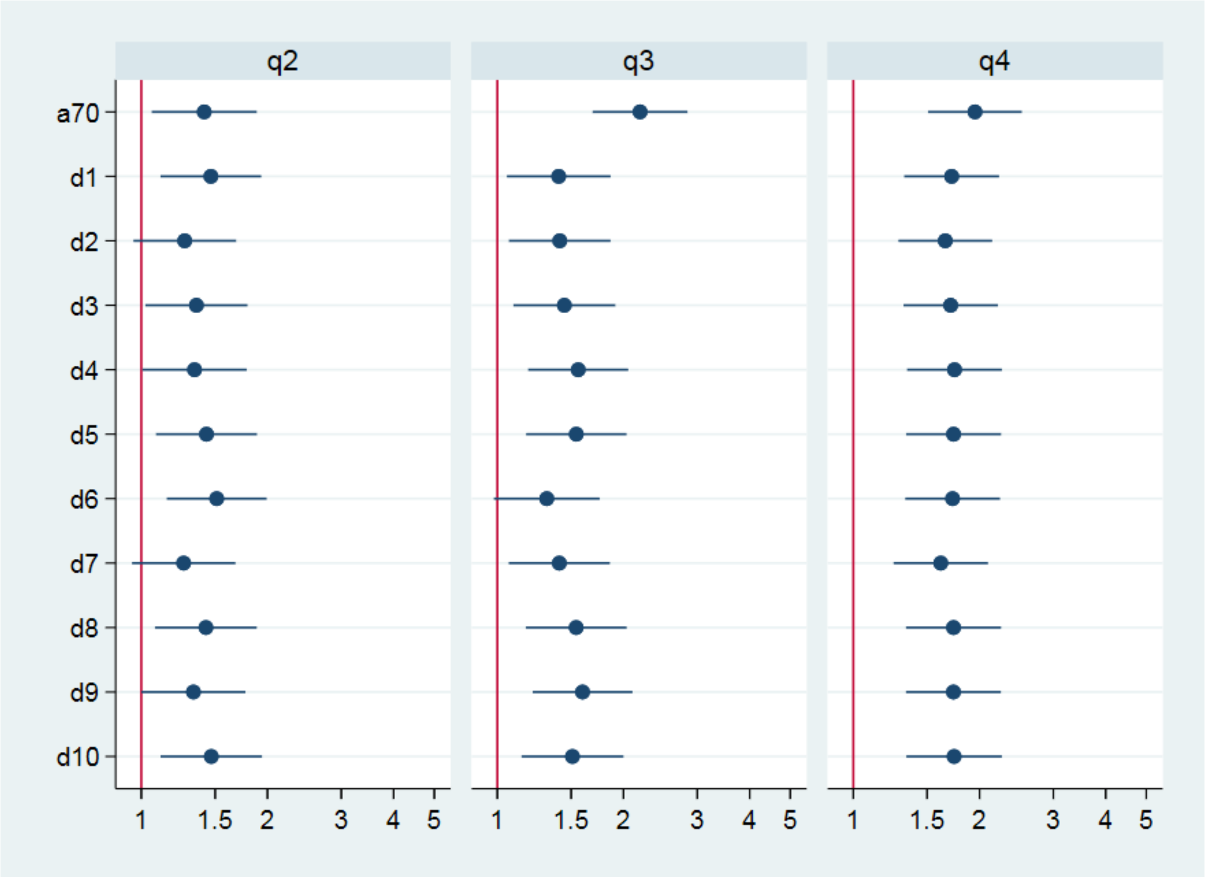

Figure 3. Sensitivity analyses of employment durations and exposure-response associations between bladder cancer mortality and lifetime cumulative exposure to N-Nitrosamines sum score

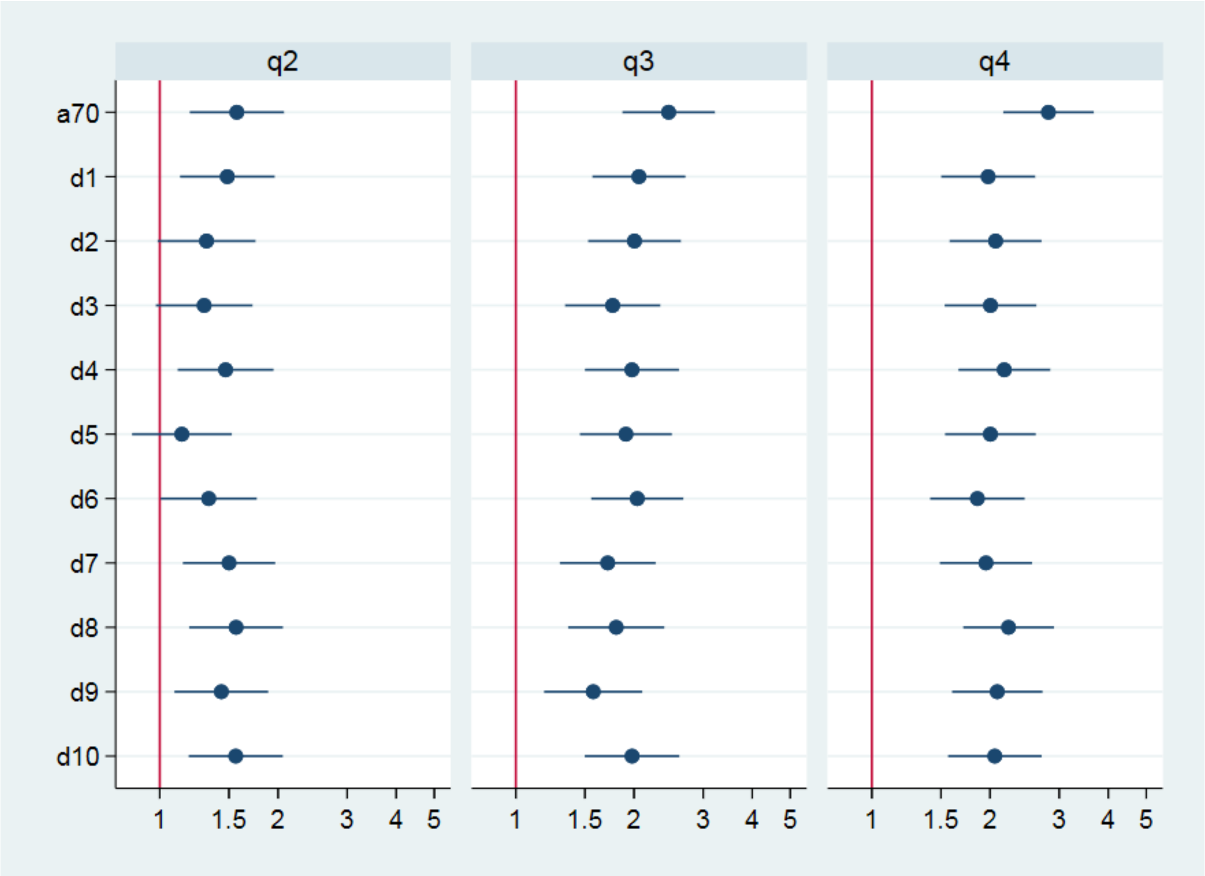

Figure 4. Sensitivity analyses of employment durations and exposure-response associations between bladder cancer mortaltiy and lifetime cumulative exposure to NDMA

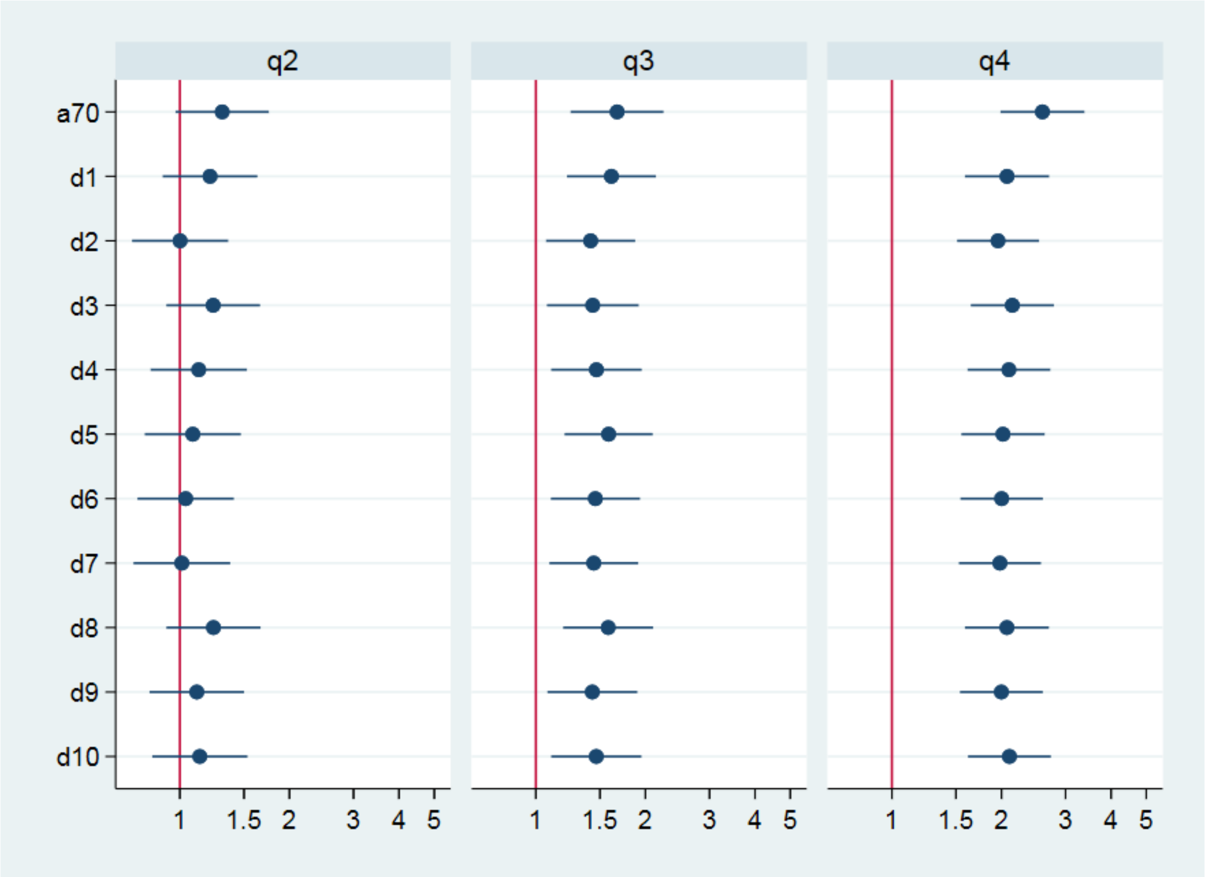

Figure 5. Sensitivity analyses of employment durations and exposure-response associations between bladder cancer mortality and lifetime cumulative exposure to NMor

LUNG

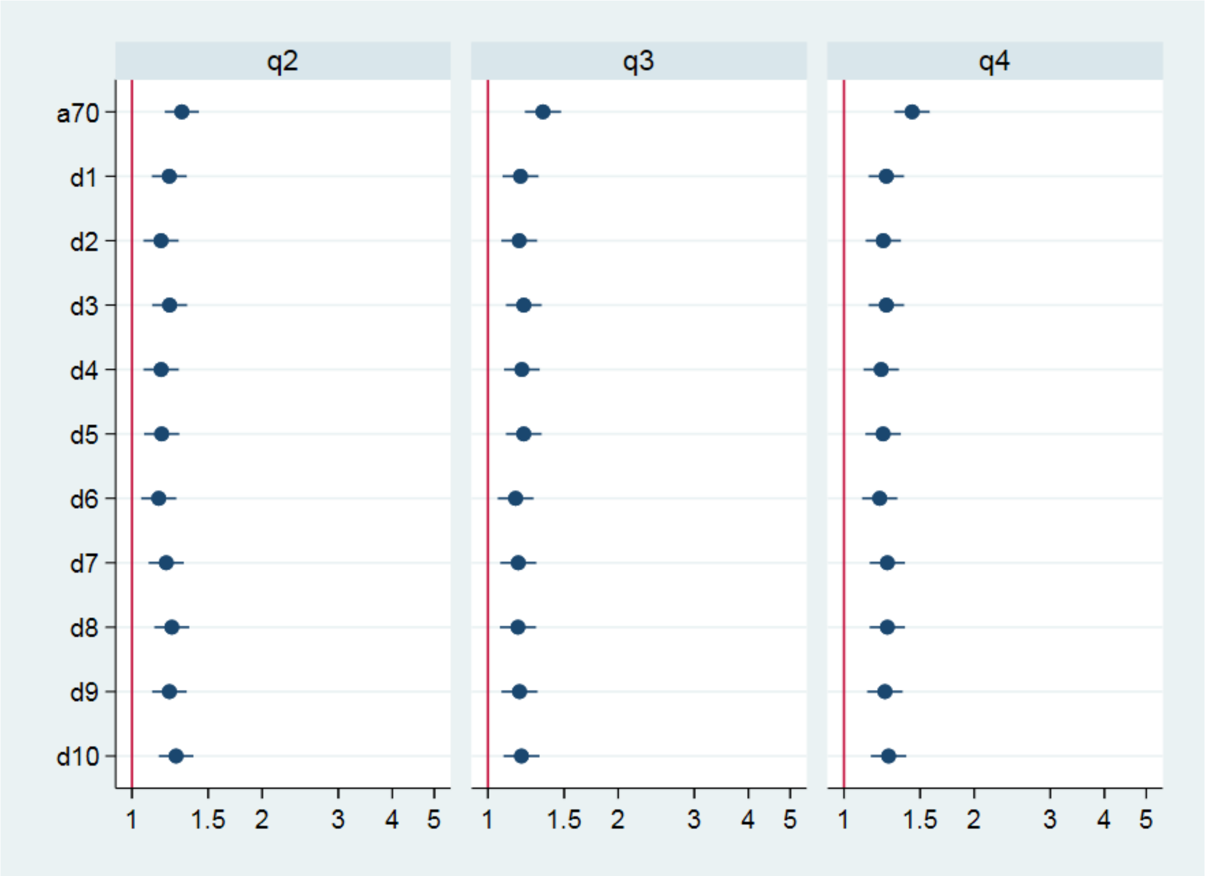

Figure 6. Sensitivity analyses of employment durations and exposure-response associations between lung cancer mortality and lifetime cumulative exposure to rubber dust

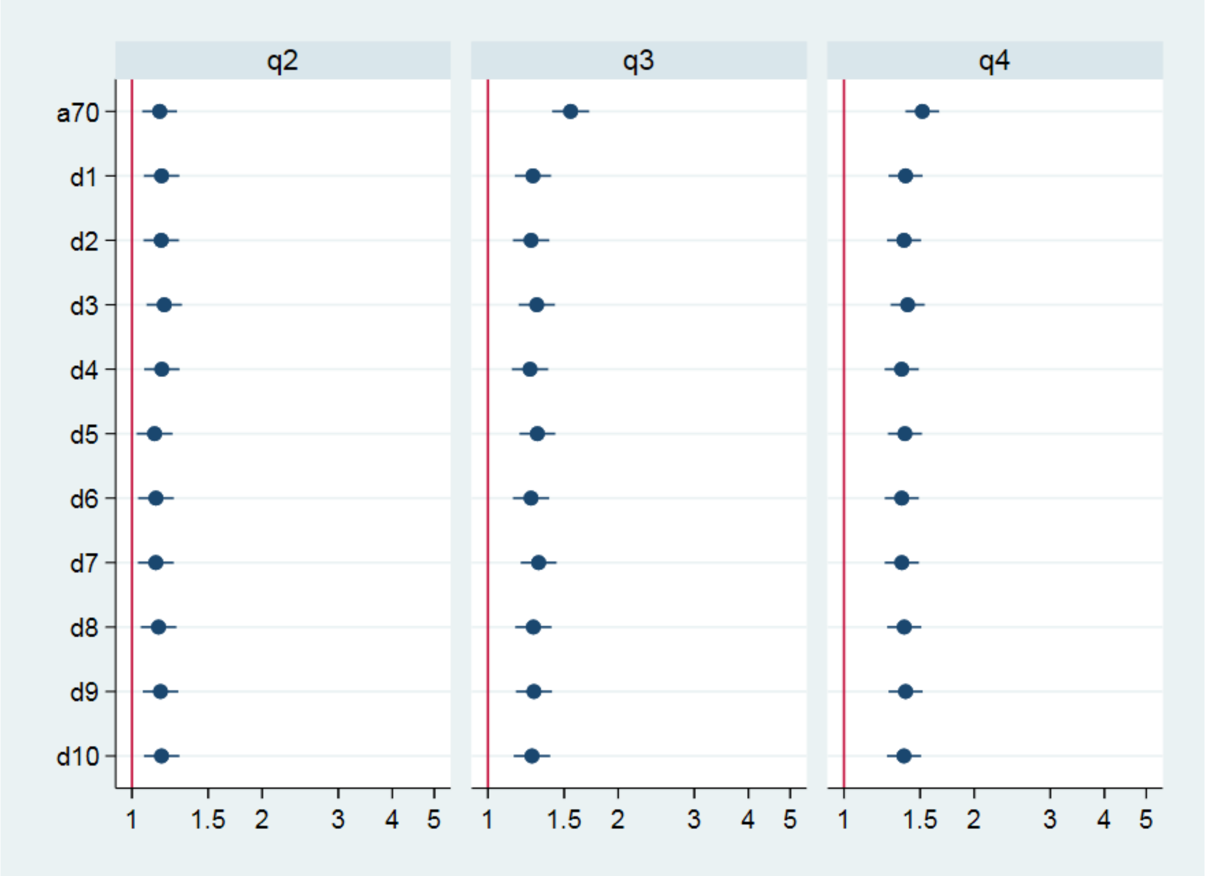

Figure 7. Sensitivity analyses of employment durations and exposure-response associations between lung cancer mortality and lifetime cumulative exposure to rubber fumes

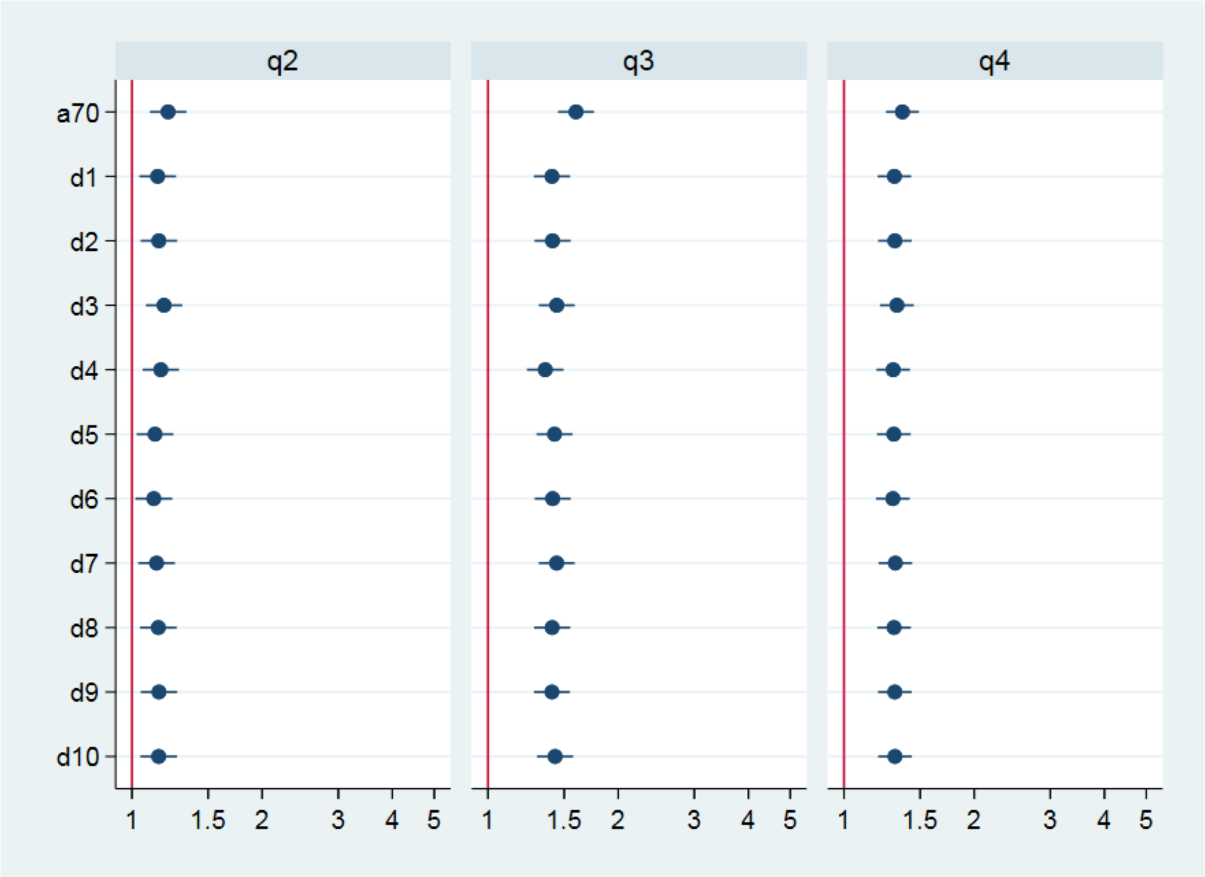

Figure 8. Sensitivity analyses of employment durations and exposure-response associations between lung cancer mortality and lifetime cumulative exposure to N-Nitrosamines sum score

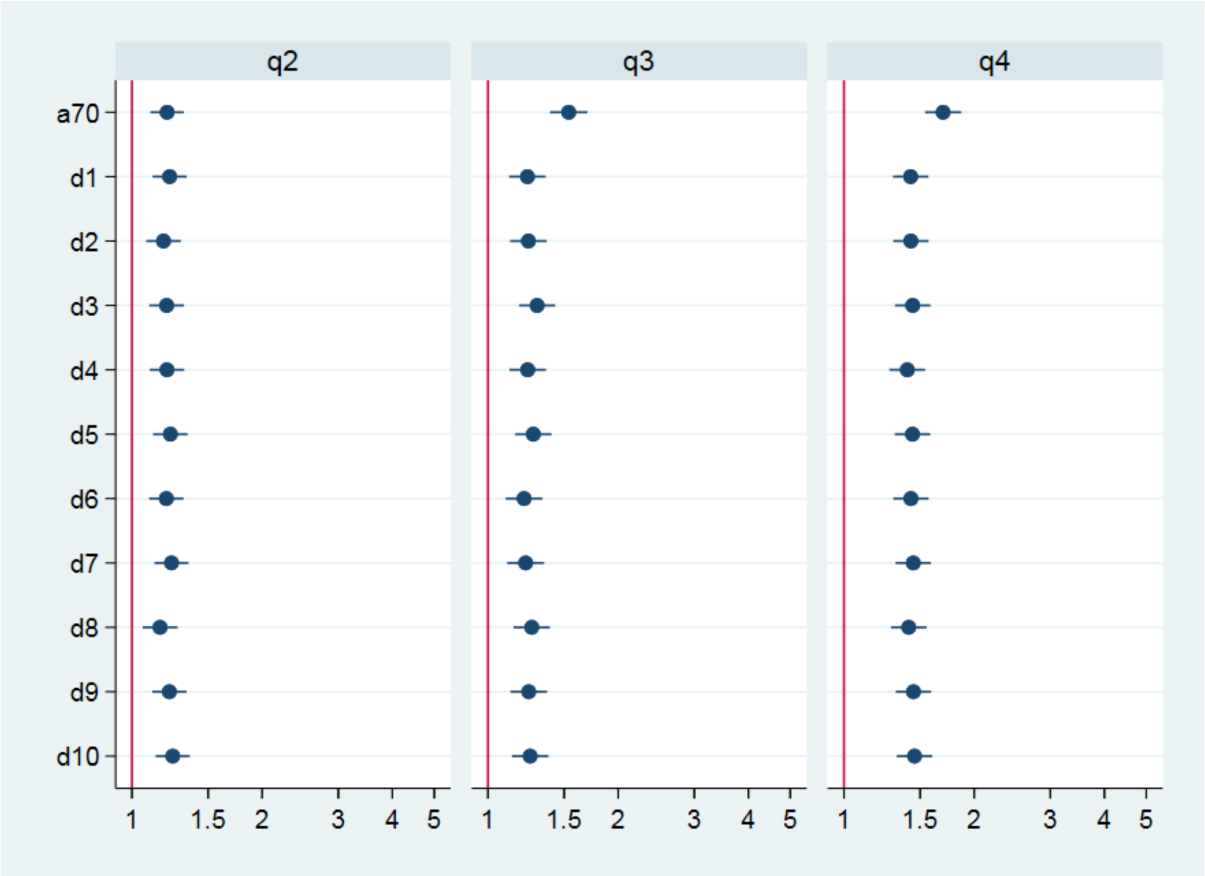

Figure 9. Sensitivity analyses of employment durations and exposure-response associations between lung cancer mortality and lifetime cumulative exposure to NDMA

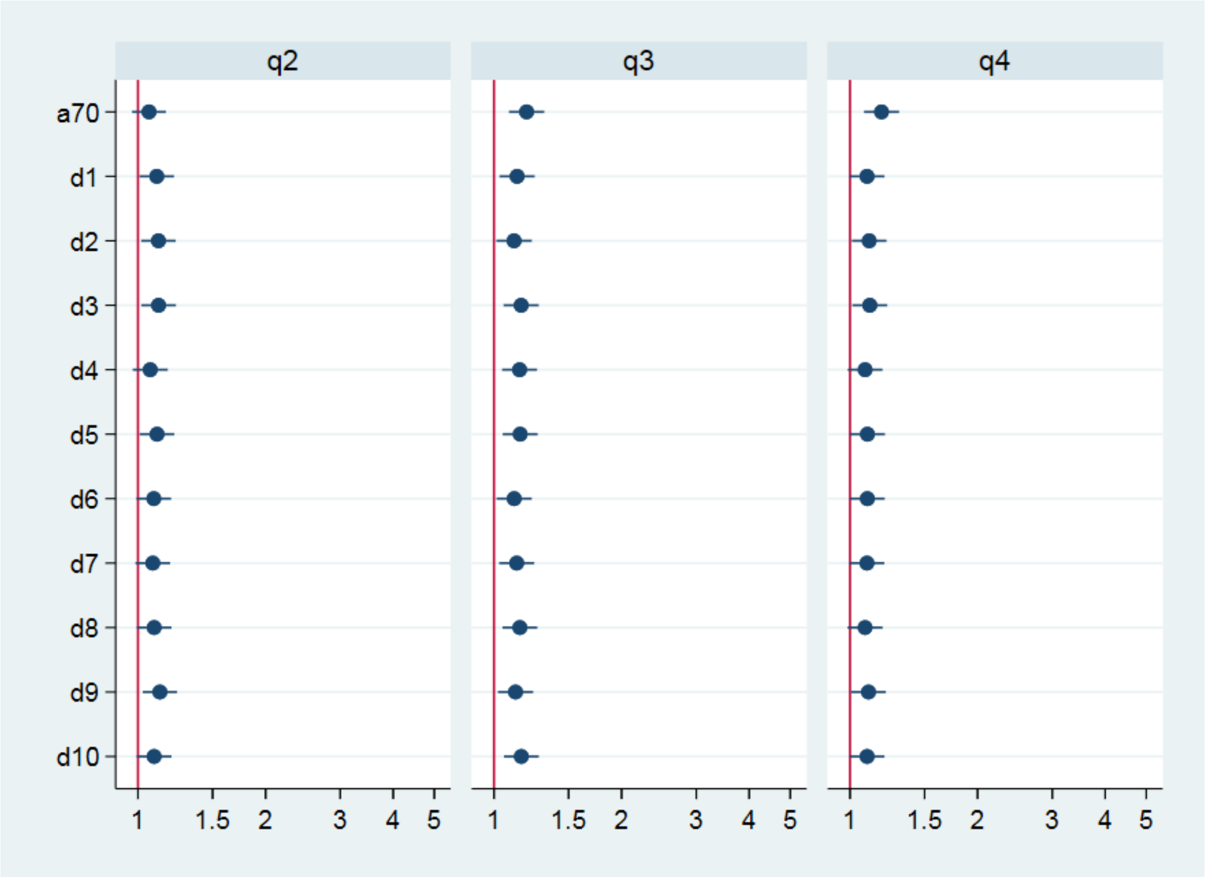

Figure 10. Sensitivity analyses of employment durations and exposure-response associations between lung cancer mortality and lifetime cumulative exposure to NMor

STOMACH

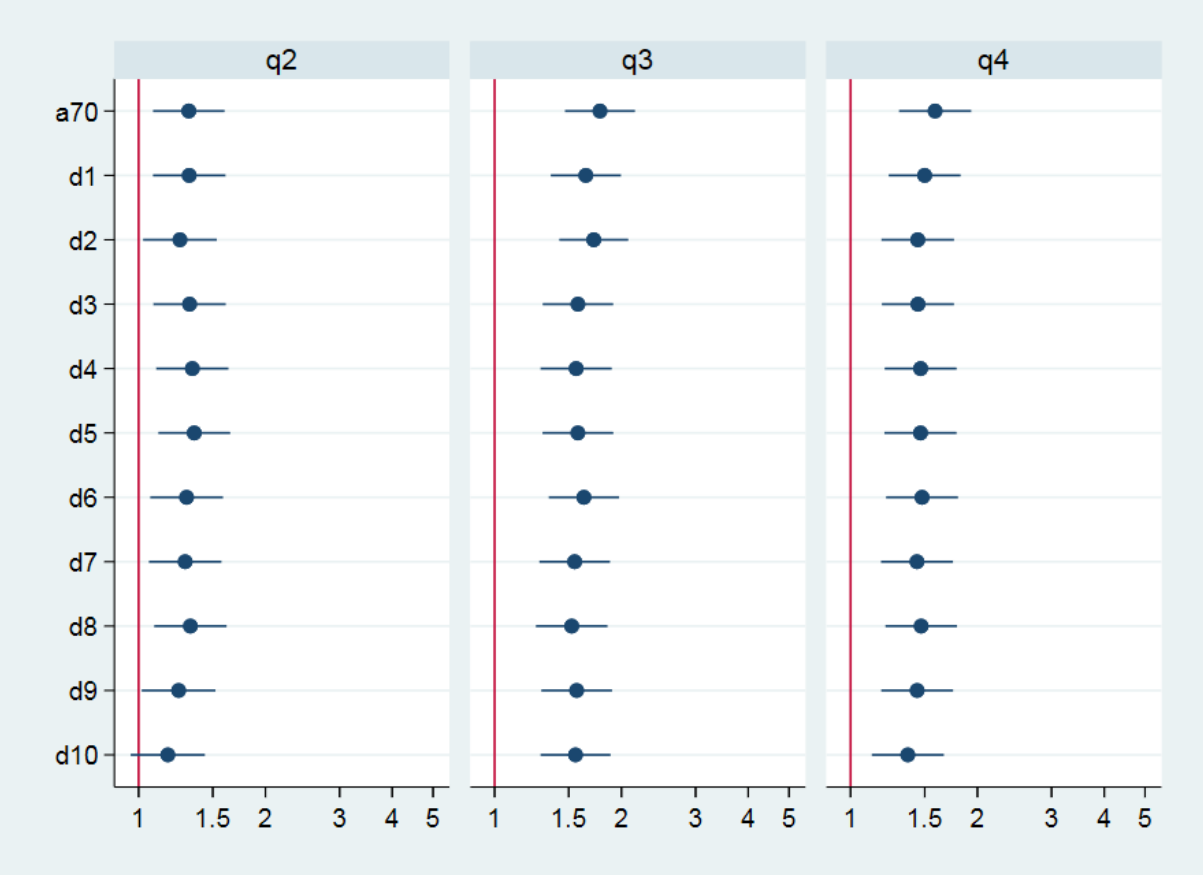

Figure 11. Sensitivity analyses of employment durations and exposure-response associations between stomach cancer mortality and lifetime cumulative exposure to rubber dust

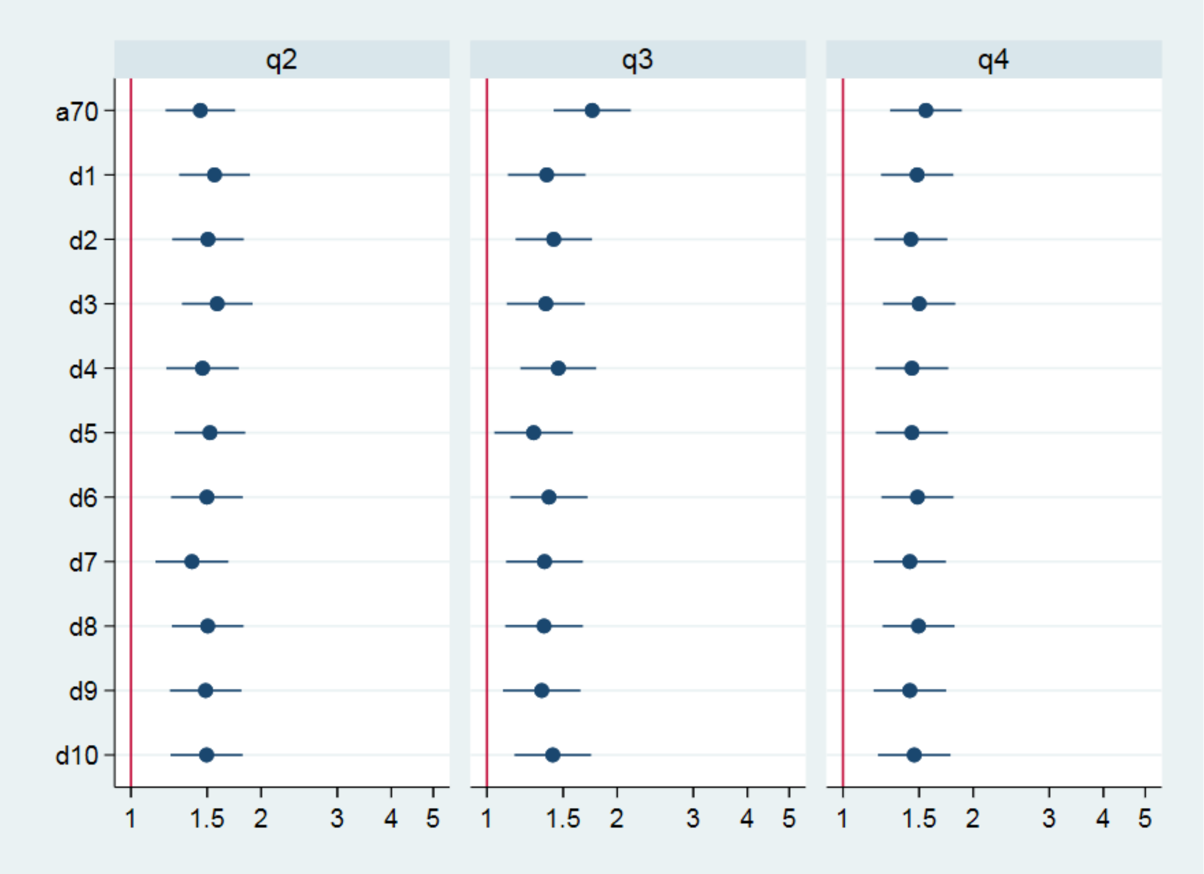

Figure 12. Sensitivity analyses of employment durations and exposure-response associations between stomach cancer mortality and lifetime cumulative exposure to rubber fumes

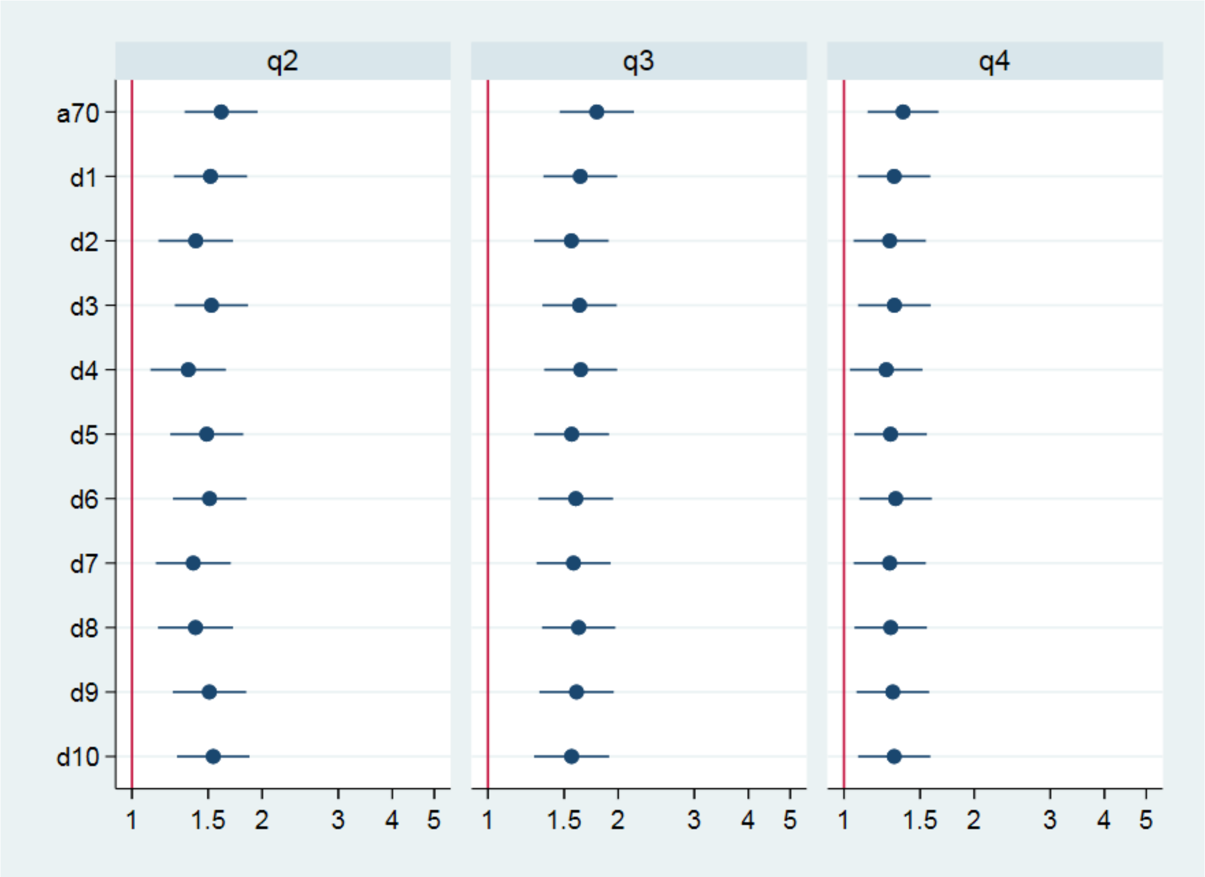

Figure 13. Sensitivity analyses of employment durations and exposure-response associations between stomach cancer mortality and lifetime cumulative exposure to N-Nitrosamines sum score

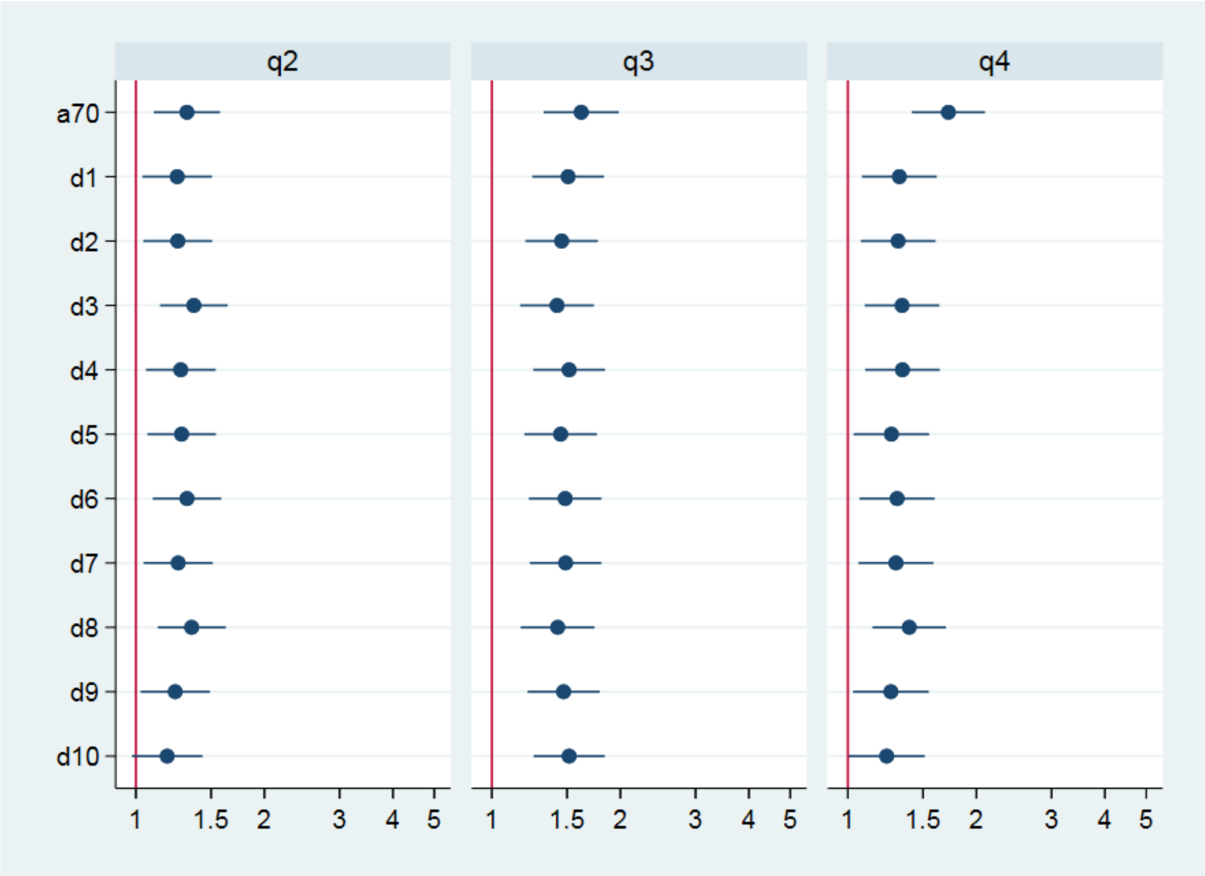

Figure 14. Sensitivity analyses of employment durations and exposure-response associations between stomach cancer mortality and lifetime cumulative exposure to NDMA

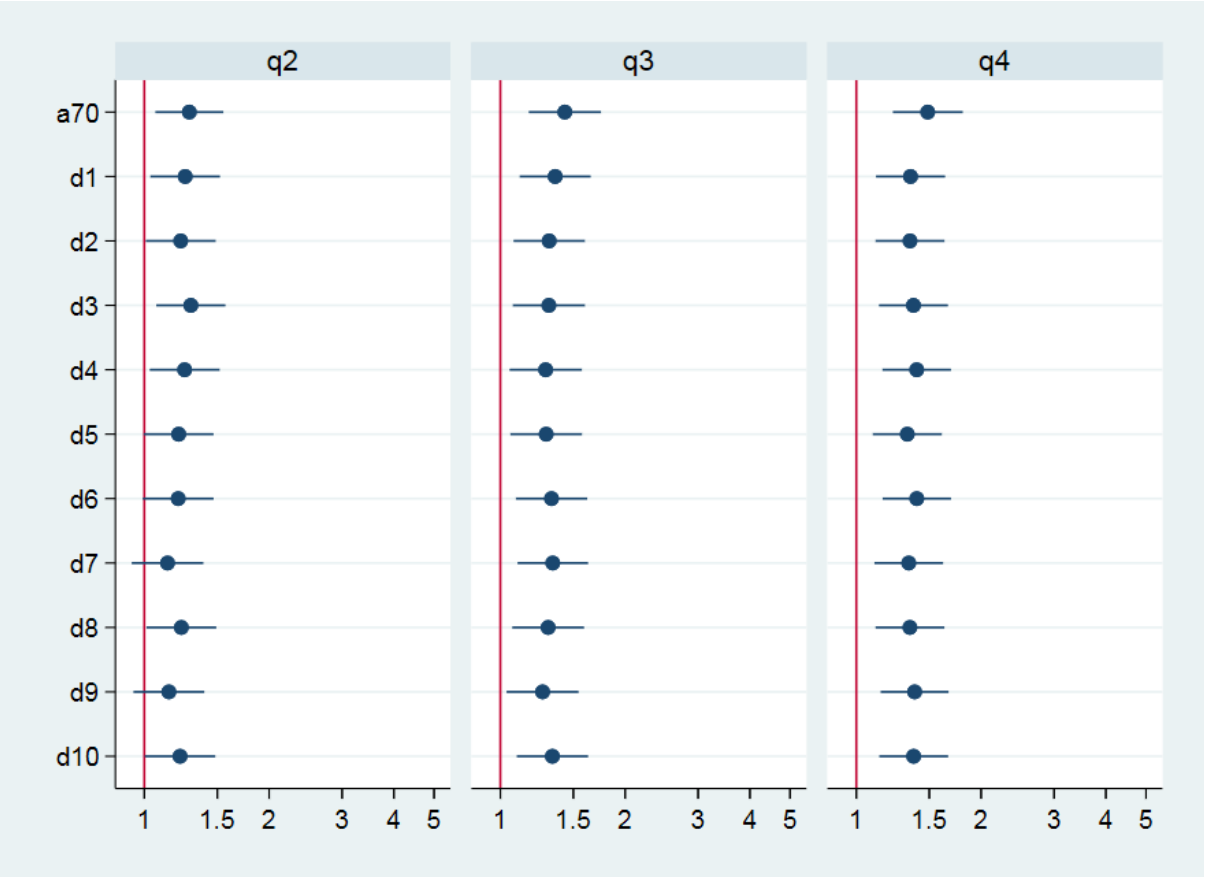

Figure 15. Sensitivity analyses of employment durations and exposure-response associations between stomach cancer mortality and lifetime cumulative exposure to NMor

MYELOMA

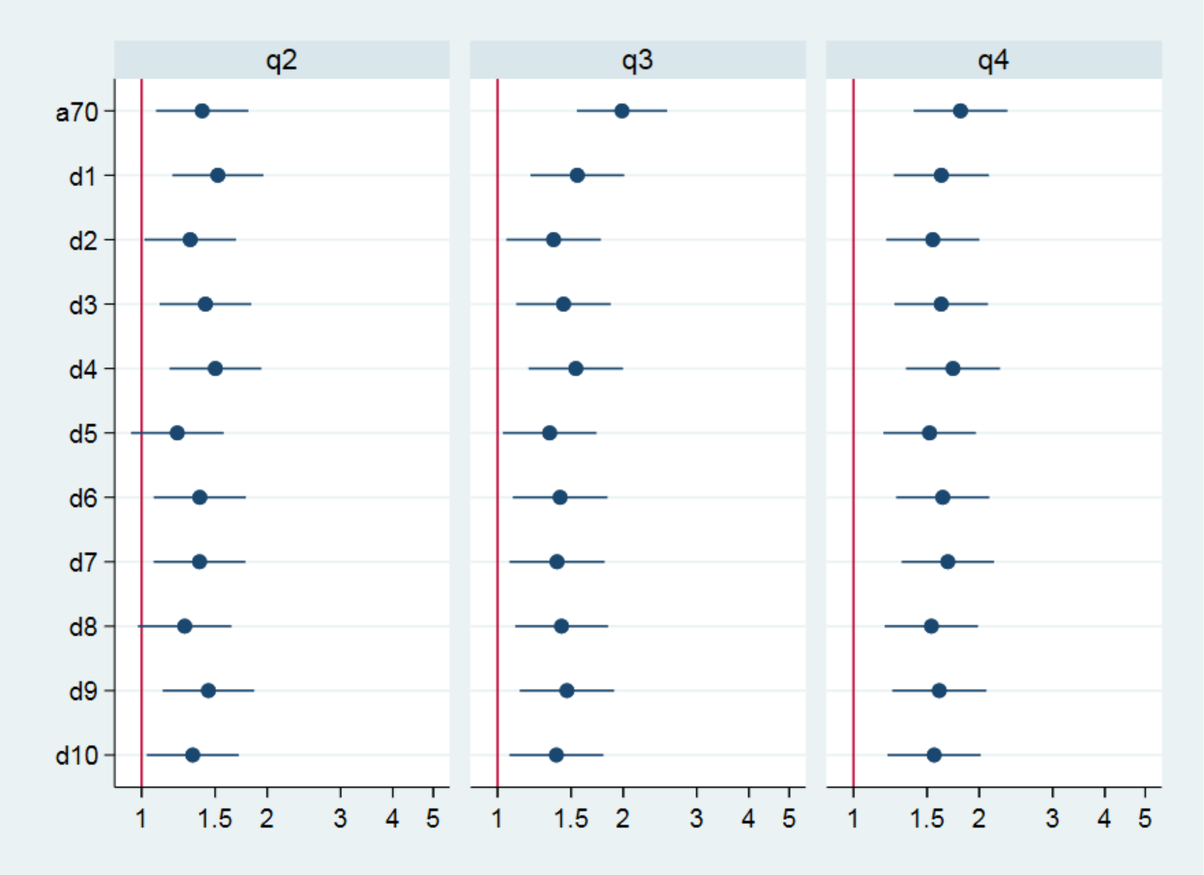

Figure 16. Sensitivity analyses of employment durations and exposure-response associations between multiple myeloma mortality and lifetime cumulative exposure to rubber dust

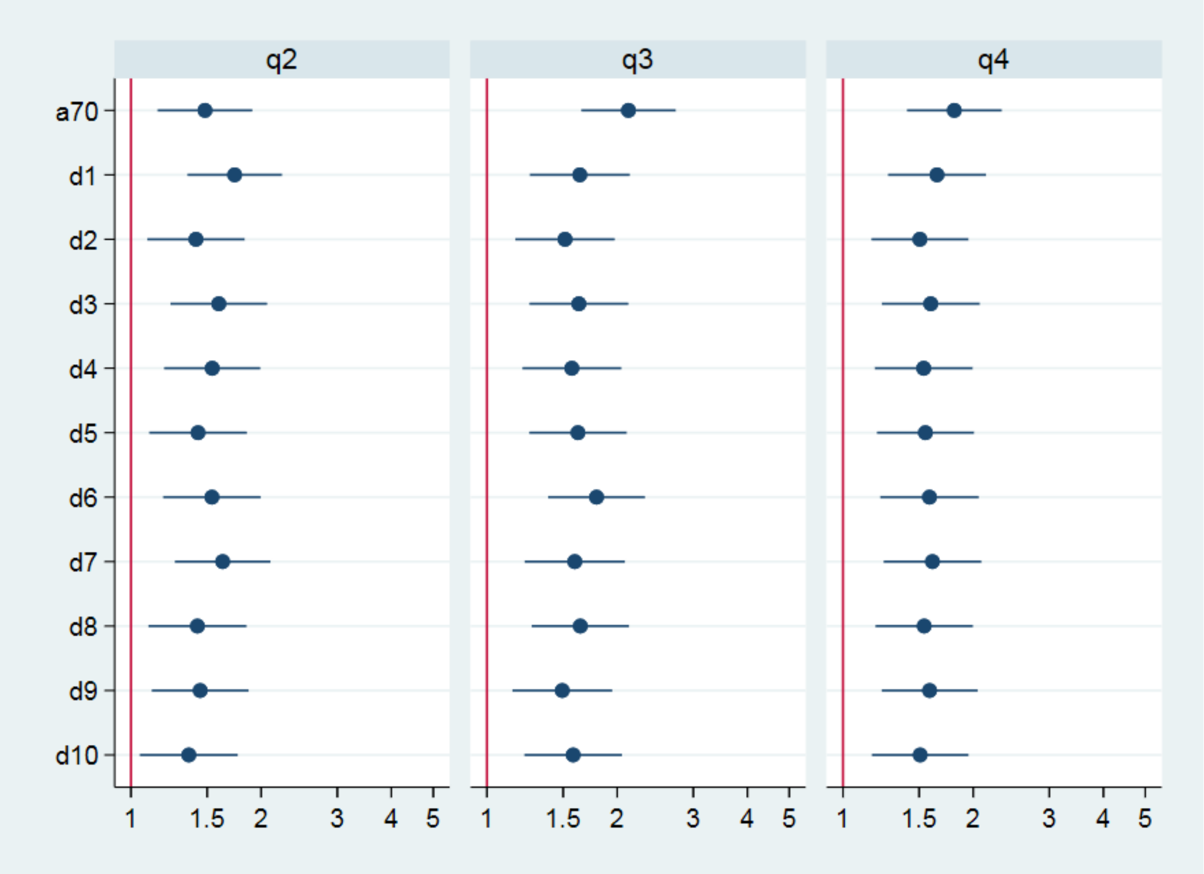

Figure 17. Sensitivity analyses of employment durations and exposure-response associations between multiple myeloma mortality and lifetime cumulative exposure to rubber fumes

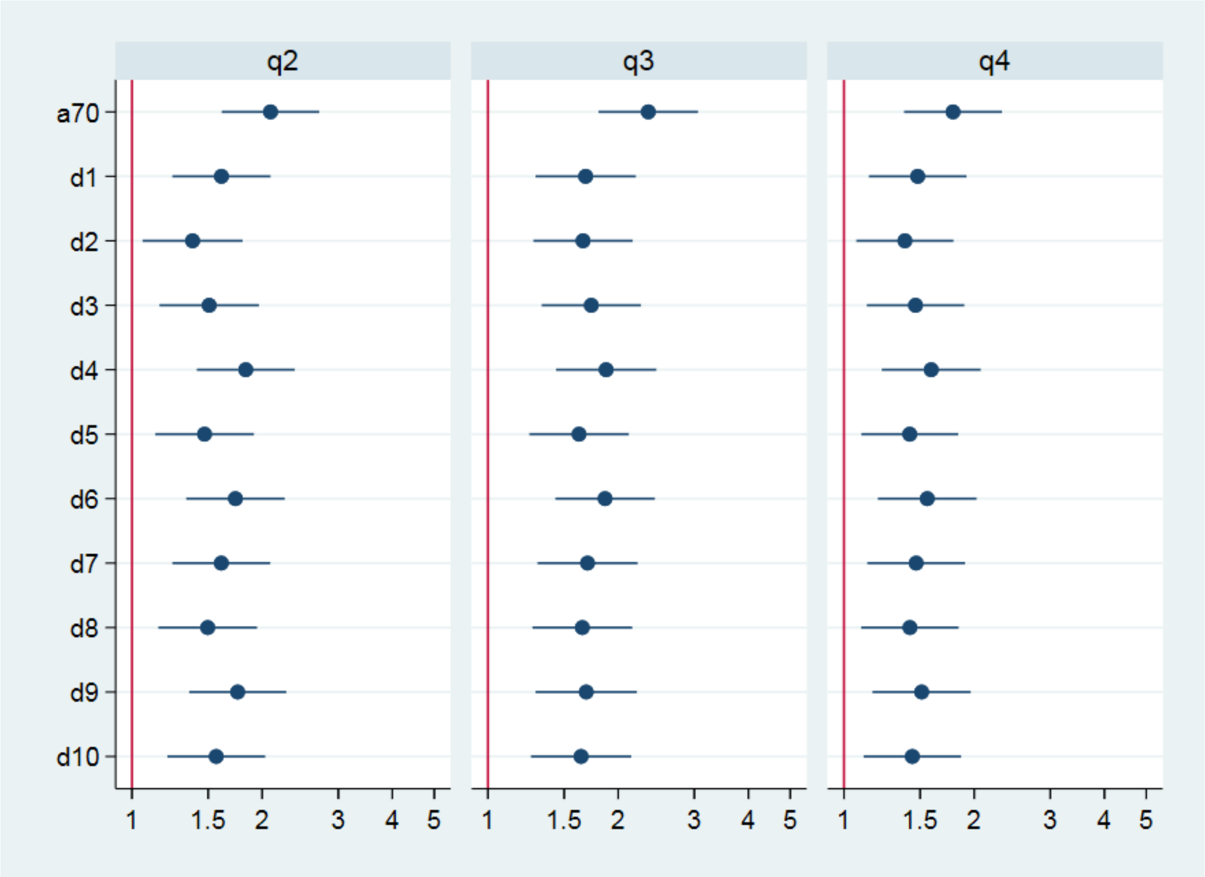

Figure 18. Sensitivity analyses of employment durations and exposure-response associations between multiple myeloma mortality and lifetime cumulative exposure to N-Nitrosamines sum score

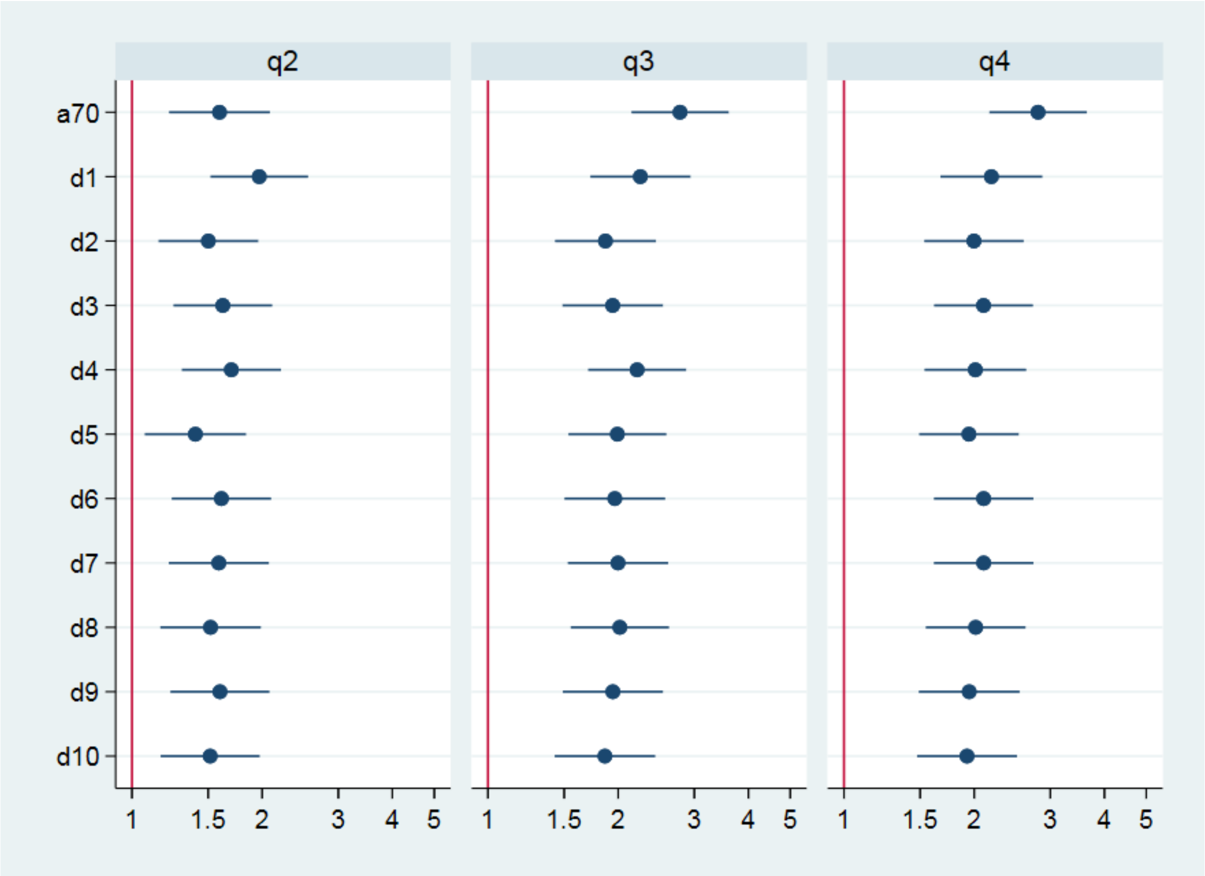

Figure 19. Sensitivity analyses of employment durations and exposure-response associations between multiple myeloma mortality and lifetime cumulative exposure to NDMA

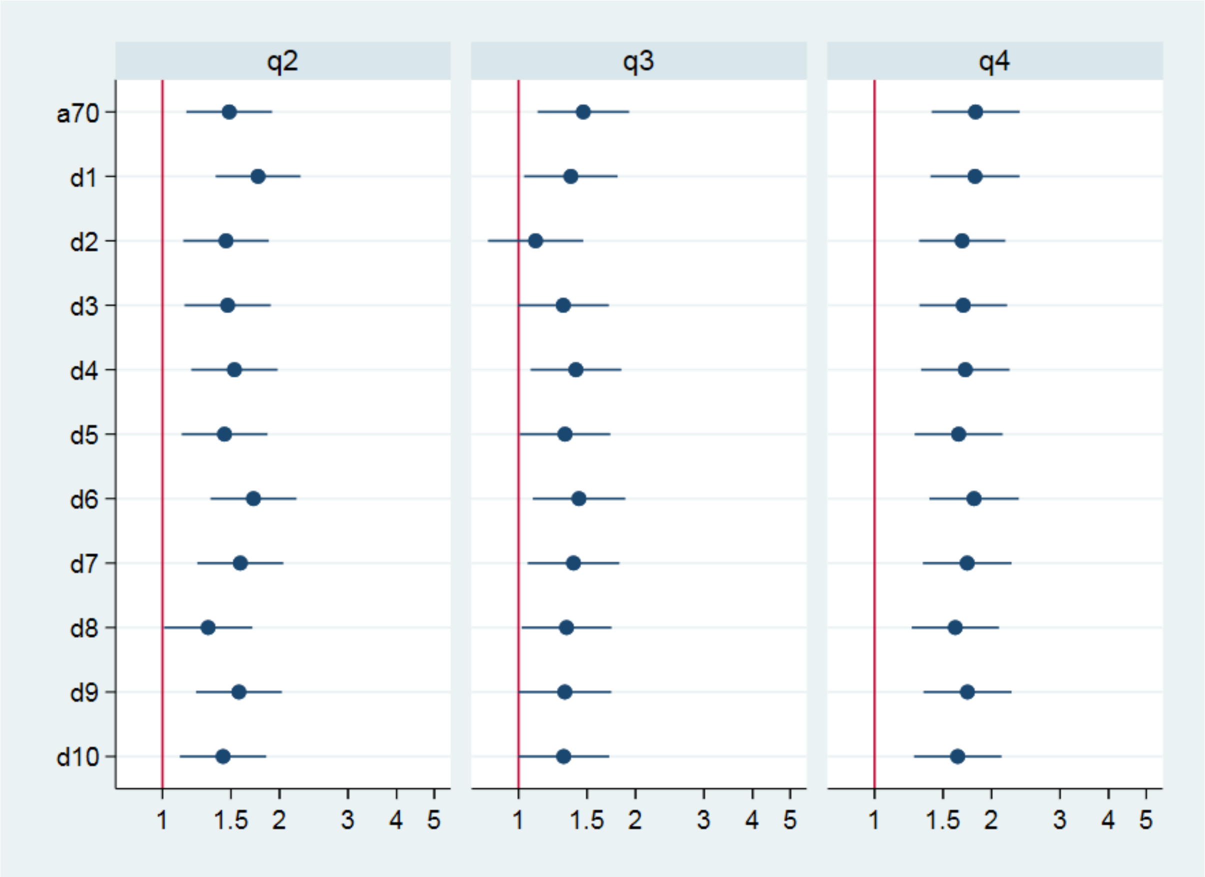

Figure 20. Sensitivity analyses of employment durations and exposure-response associations between multiple myeloma mortality and lifetime cumulative exposure to NMor

LEUKAEMIA

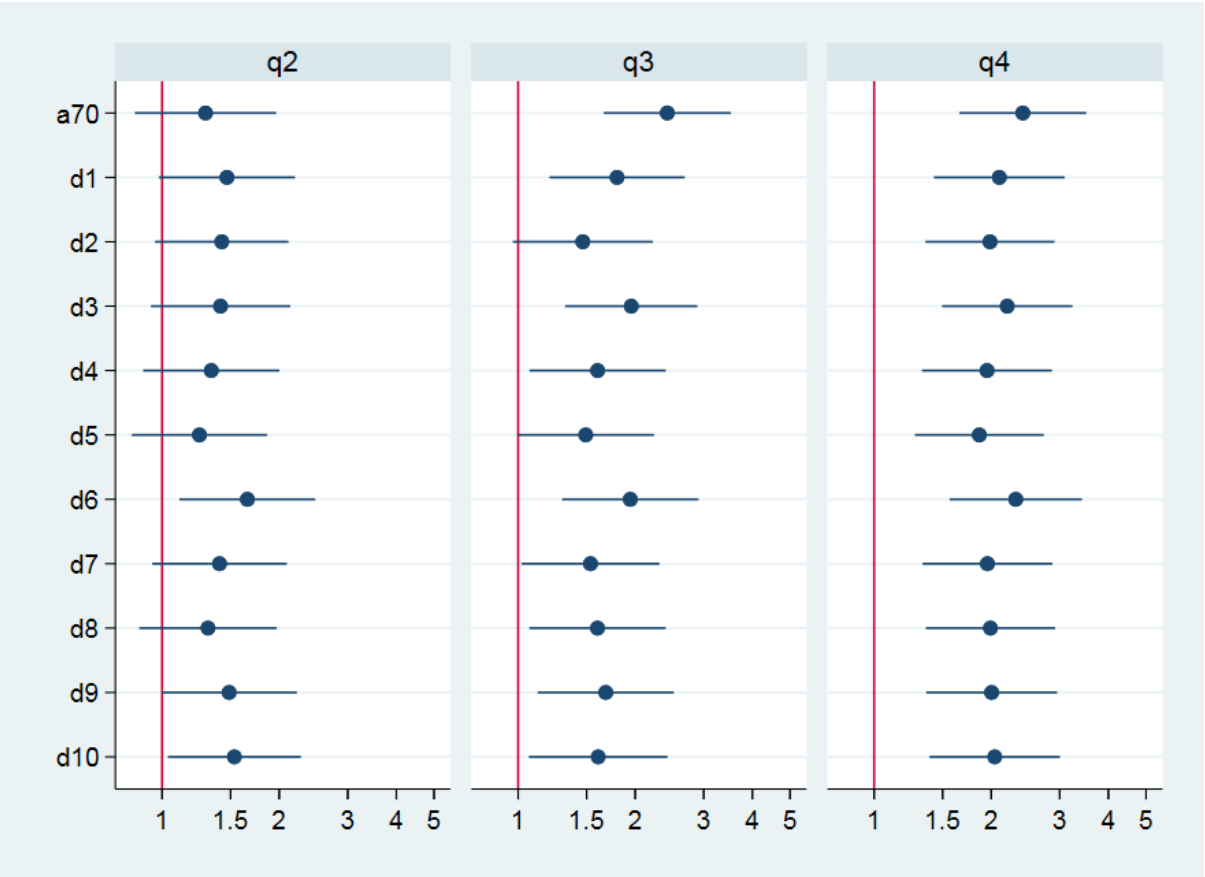

Figure 21. Sensitivity analyses of employment durations and exposure-response associations between leukaemia mortality and lifetime cumulative exposure to rubber dust

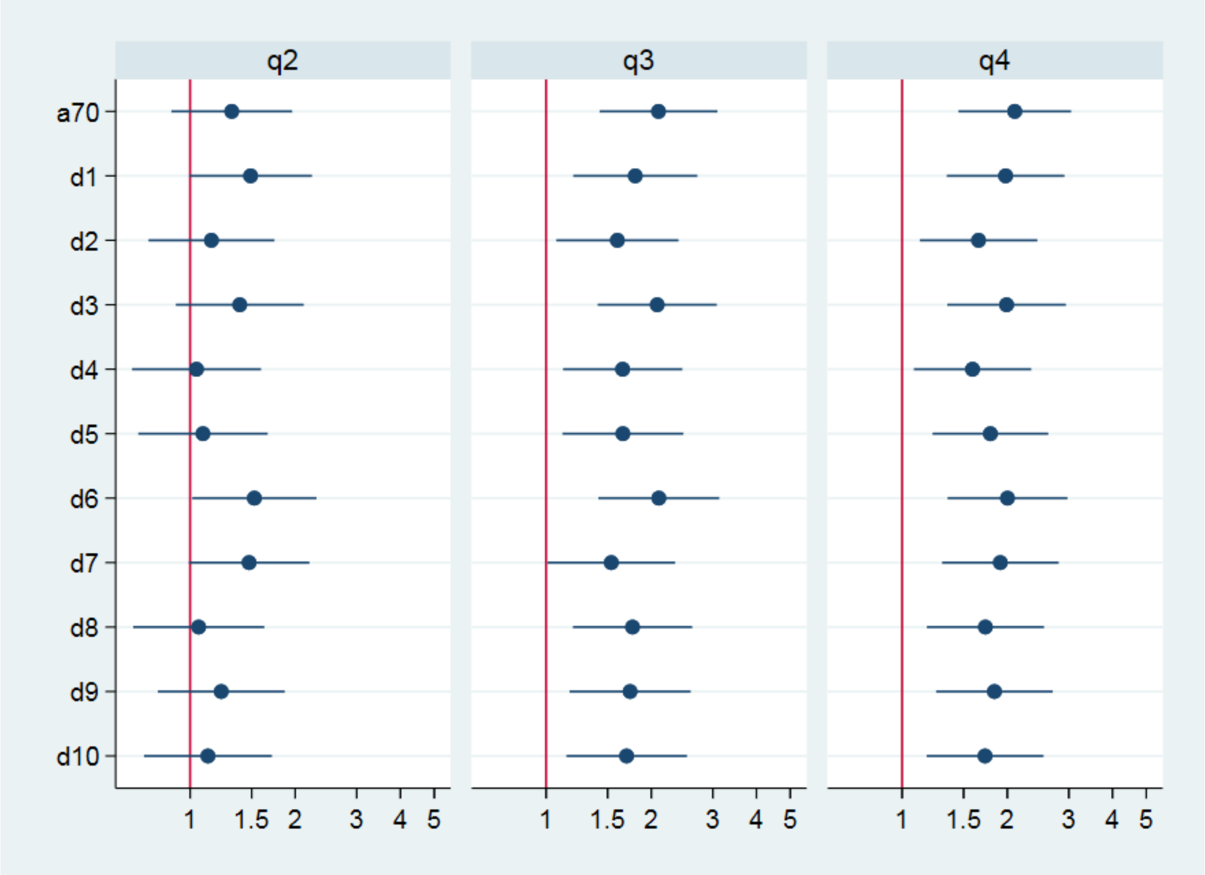

Figure 22. Sensitivity analyses of employment durations and exposure-response associations between leukaemia mortality and lifetime cumulative exposure to rubber fumes

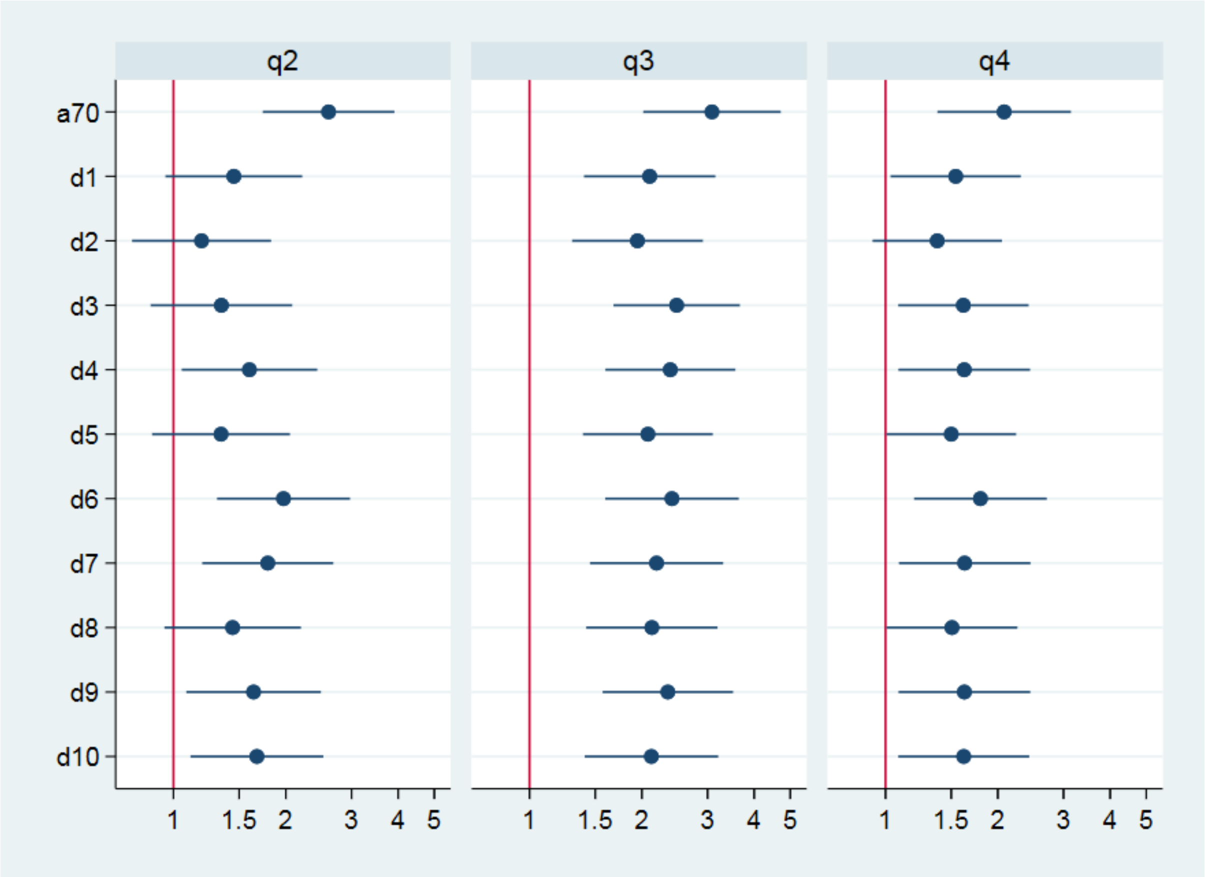

Figure 23. Sensitivity analyses of employment durations and exposure-response associations between leukaemia mortality and lifetime cumulative exposure to N-Nitrosamines

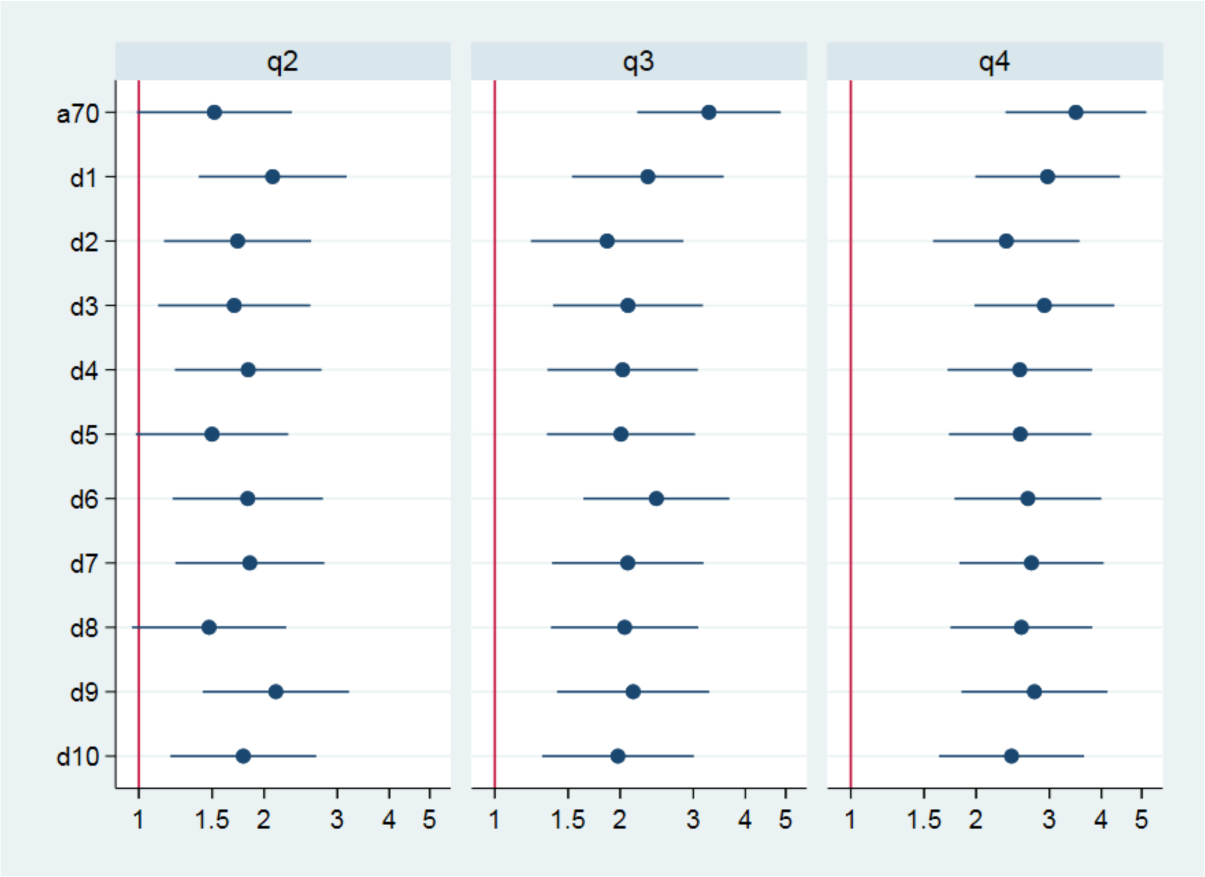

Figure 24. Sensitivity analyses of employment durations and exposure-response associations between leukaemia mortality and lifetime cumulative exposure to NDMA

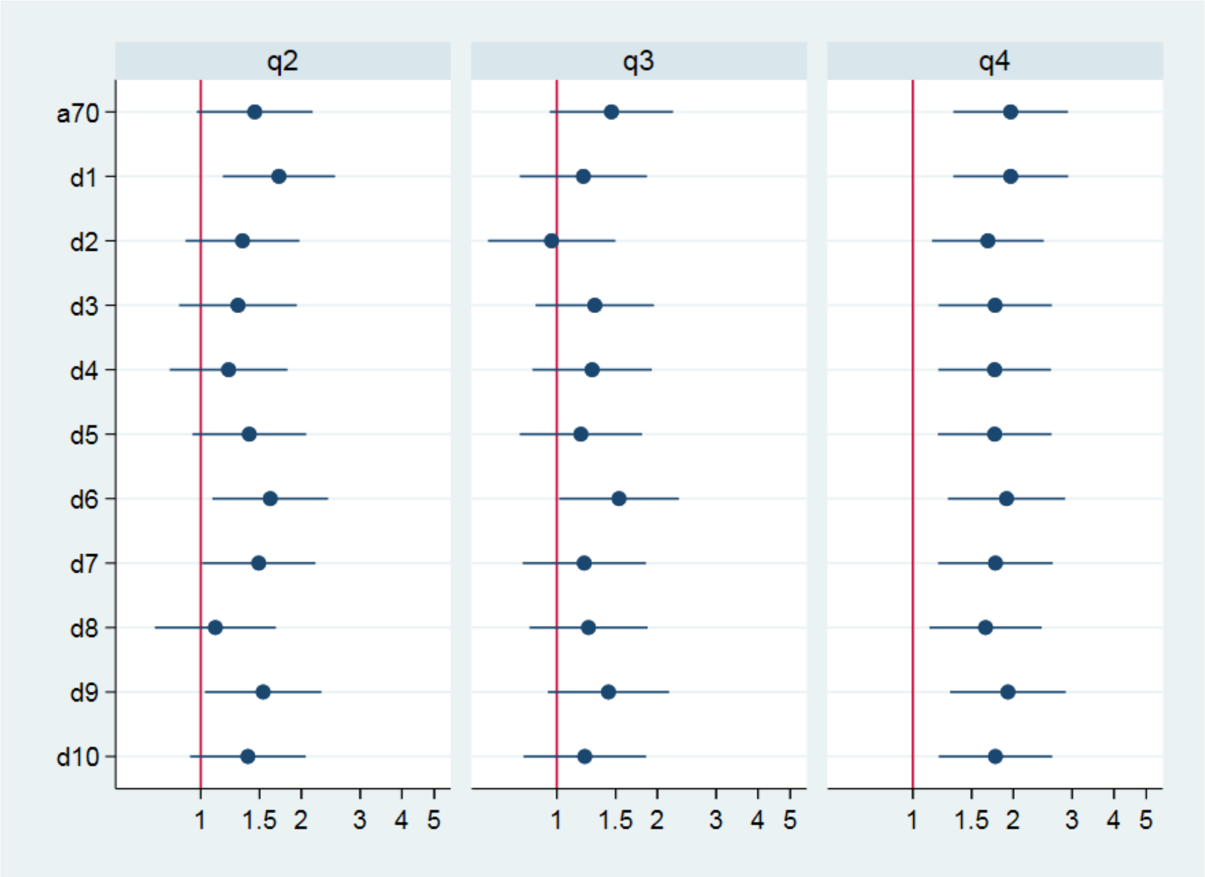

Figure 25. Sensitivity analyses of employment durations and exposure-response associations between leukaemia mortality and lifetime cumulative exposure to NMor

MALIGNANT NEOPLASMS

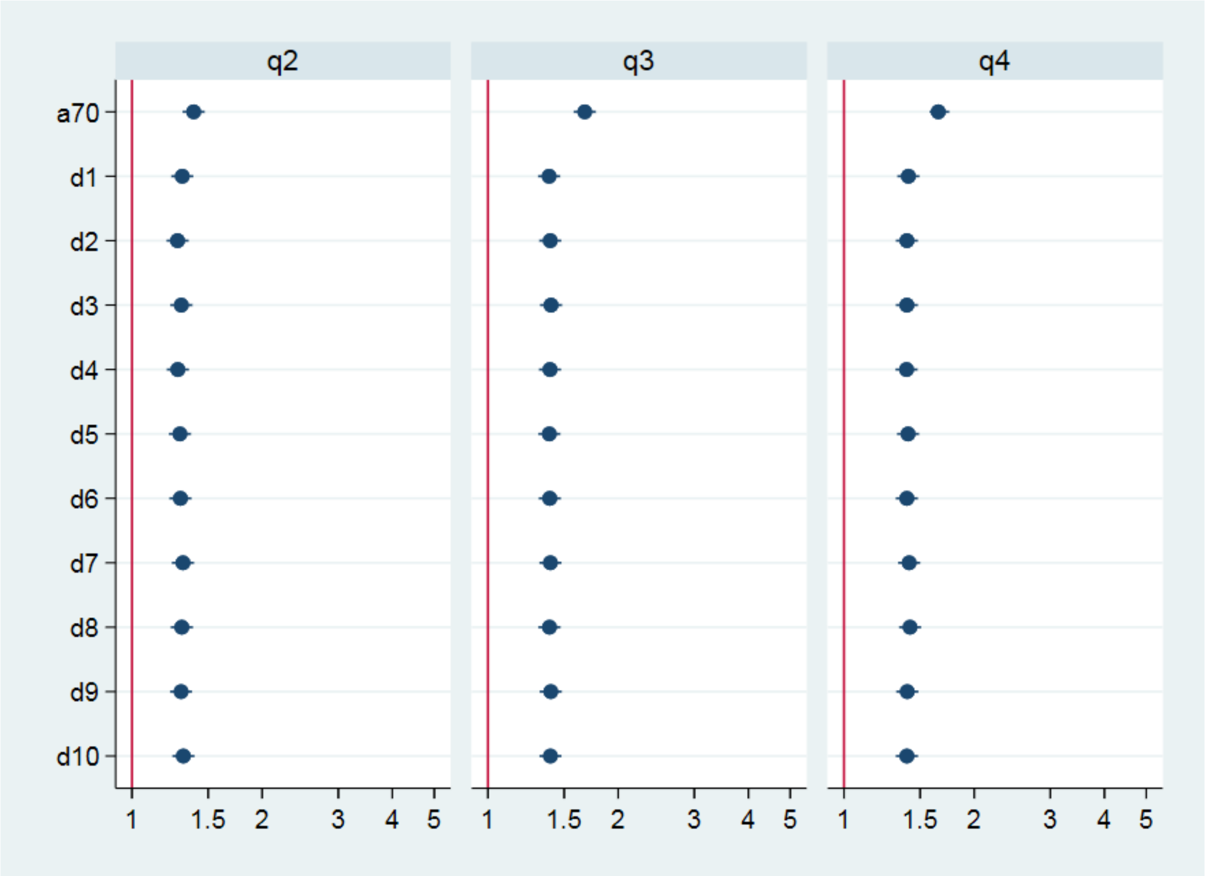

Figure 26. Sensitivity analyses of employment durations and exposure-response associations between all malignant neoplasms mortality and lifetime cumulative exposure to rubber dust

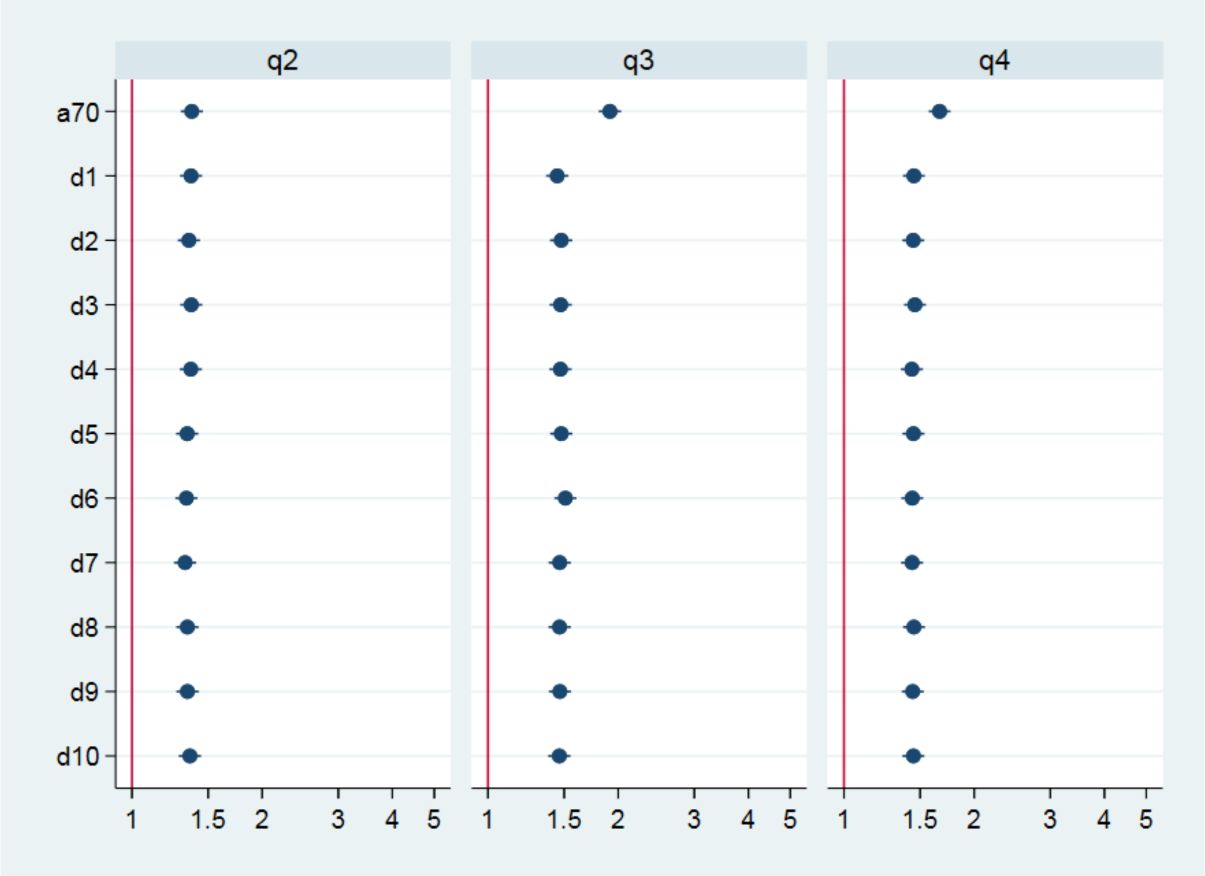

Figure 27. Sensitivity analyses of employment durations and exposure-response associations between all malignant neoplasms mortality and lifetime cumulative exposure to rubber fumes

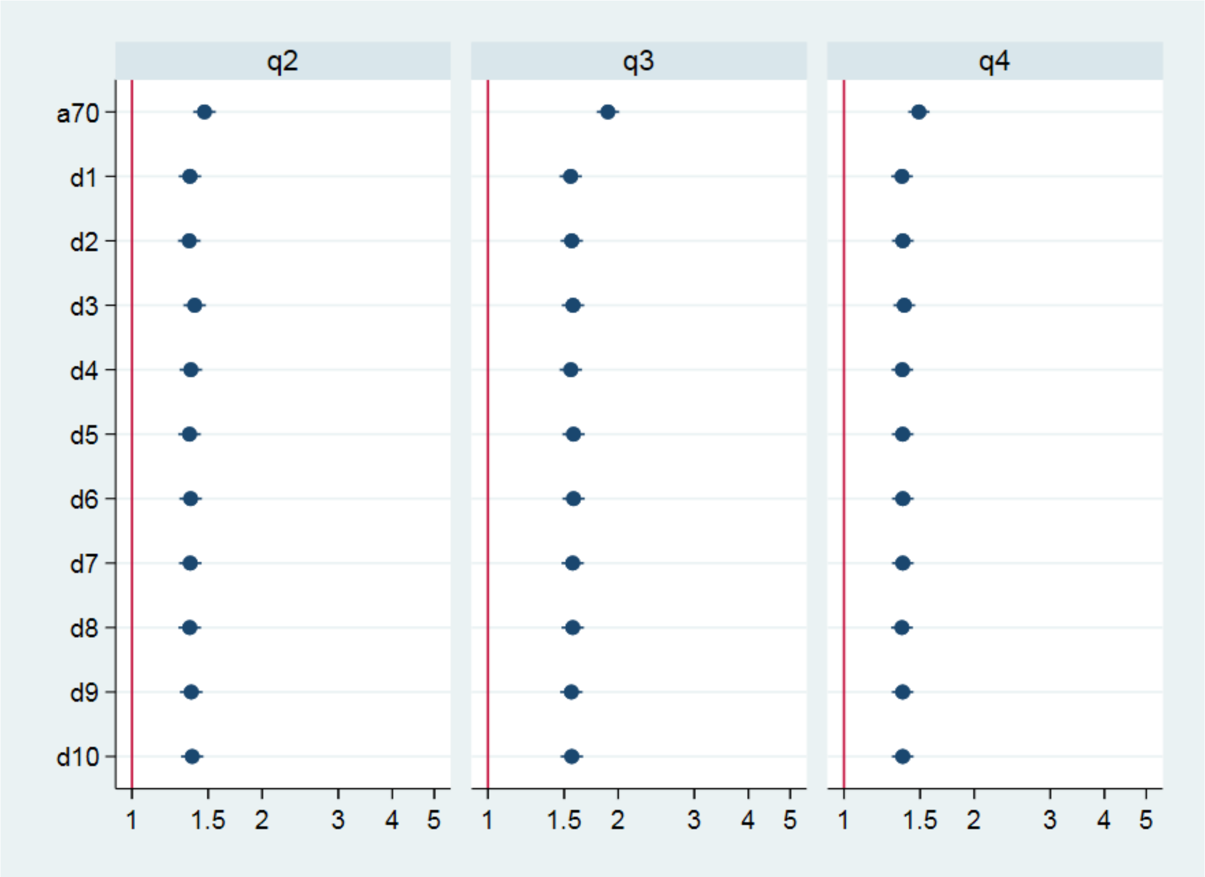

Figure 28. Sensitivity analyses of employment durations and exposure-response associations between all malignant neoplasms mortality and lifetime cumulative exposure to N-Nitrosamines sum score

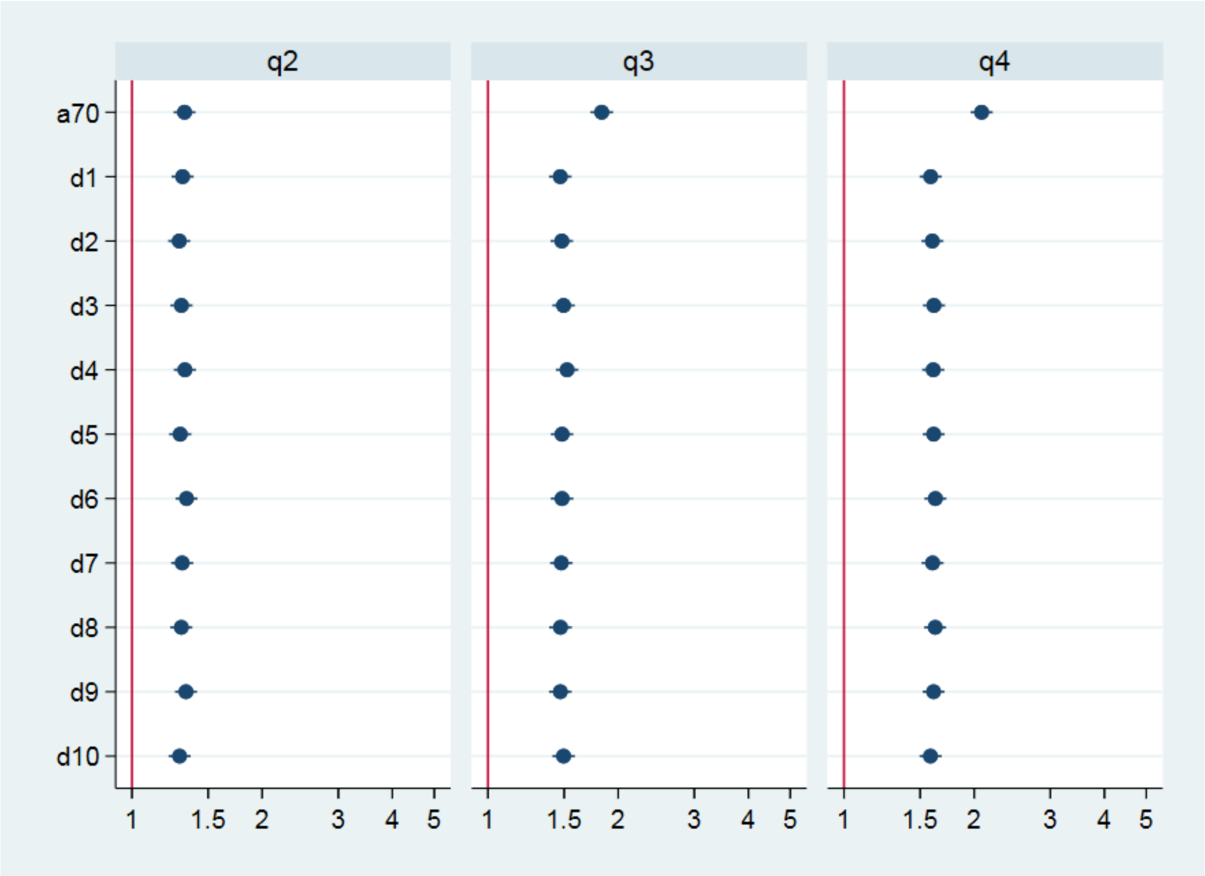

Figure 29. Sensitivity analyses of employment durations and exposure-response associations between all malignant neoplasms mortality and lifetime cumulative exposure to NDMA

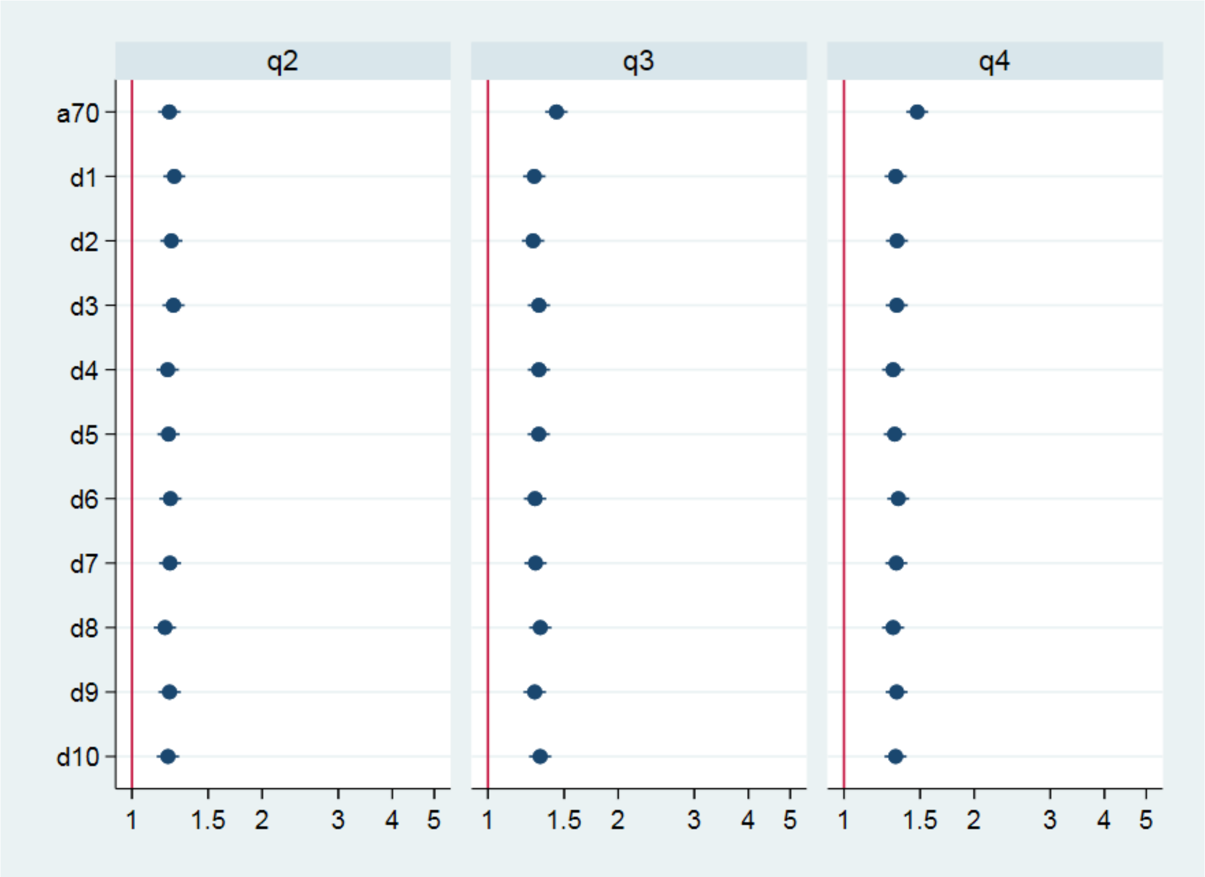

Figure 30. Sensitivity analyses of employment durations and exposure-response associations between all malignant neoplasms mortality and lifetime cumulative exposure to NMor
